# ABA promotes fatty acid biosynthesis and transport to boost arbuscular mycorrhizal symbiosis in apple roots

**DOI:** 10.1016/j.xplc.2025.101426

**Published:** 2025-06-24

**Authors:** Shan Jing, Mingjun Li, Chunhui Li, Chunlei Zhang, Lingcheng Zhu, Lijun Du, Yuchao Li, Xiaoyu Wei, Manrang Zhang, Baiquan Ma, Yongling Ruan, Fengwang Ma

**Affiliations:** State Key Laboratory for Crop Stress Resistance and High-Efficiency Production / Shaanxi Key Laboratory of Apple, College of Horticulture, Northwest A&F University, Yangling, Shaanxi 712100, China

**Keywords:** abscisic acid, mycorrhizal symbiosis, ABF2, lipid biosynthesis and transport, *Malus pumila* Mill

## Abstract

The roots of most land plants form symbioses with arbuscular mycorrhizal (AM) fungi. The fungus promotes nutrient uptake from the soil while receiving plant-derived photosynthates as lipids and sugars. Nutrient exchange must be regulated by both partners; however, the mechanisms underlying the regulation of lipid supply from the plant to the AM fungus remain unclear. Here, we performed a molecular study on the role of elevated abscisic acid (ABA) levels during AM fungal infection in apple (*Malus* spp.) roots. AM fungal colonization induced the expression of two ABA biosynthesis genes, *MdNCED3.1* and *MdNCED3.2*, in apple roots and increased ABA content, which promoted AM fungal growth. The effect of ABA on symbiosis was confirmed in transgenic apple roots overexpressing or silencing *MdNCED3.1* or *MdNCED3.2*. Transcriptome analysis and transgenic experiments revealed that the transcription factor MdABF2 plays a key role in ABA-mediated symbiosis during AM infection and regulates the expression of genes associated with fatty acid (FA) biosynthesis (e.g., *MdKASIII*) and transport (such as *MdSTR2*) in apple roots. Activation of these genes increased FA levels in roots and enhanced AM fungal colonization and arbuscule development. These findings identify a molecular pathway in which ABA signaling positively regulates FA biosynthesis and transport, thereby increasing lipid supply to AM fungi and promoting AM symbiosis.

## Teaser

This study reports that ABA is essential for efficient arbuscular mycorrhizal (AM) symbiosis in apple roots. AM fungal infection induces ABA biosynthesis, which in turn promotes fatty acid (FA) biosynthesis and transport via the MdABF2-mediated ABA signaling pathway. The resulting elevation in FA levels in plant roots supplies a primary carbon source for fungal growth, thereby enhancing AM symbiosis.

## Introduction

Arbuscular mycorrhiza (AM) is a widespread symbiotic interaction formed between 80% to 90% of land plant species and *Glomeromycotina* fungi (Gadkar et al., 2001; Smith and Smith, 2011). From ecological and agronomic perspectives, this mutualistic symbiosis has received substantial attention because AM enhances crop yield and quality by modifying mineral nutrient uptake, stress resistance, and carbon and nutrient distribution in plants (Smith and Smith, 2011; Bennett and Groten, 2022; Shi et al., 2023; Wang et al., 2024). Typically, mutualistic symbioses involve the exchange of nutrient currencies that confer adaptive advantages while potentially incurring costs. Therefore, symbionts evolve regulatory mechanisms to prevent excessive exploitation by their trading partner (Kiers et al., 2011; Bennett and Groten, 2022). An optimal growth system requires a balanced transaction. This raises the question of what the AM fungus contributes to the plant and what costs the plant incurs to support the AM fungus.

The symbiotic relationship between AM fungi and host plants is based on mutual benefit, whereby the fungi provide nutrients to the plant in exchange for carbon necessary for their growth. AM fungi depend on fixed carbon from plants as their primary food source, with plants allocating a portion of their photoassimilates, in the form of carbohydrates and lipids, to the fungus (Jiang et al., 2017; Luginbuehl et al., 2017). Prior detailed ^13C-^labeling and nuclear magnetic resonance tracing studies suggested that hexoses are the primary forms of carbon transferred from plants to fungi (Shachar-Hill et al., 1995). It was previously believed that plants provided only sugars to fungi and that AM fungi utilized these sugars as precursors for lipid biosynthesis (Pfeffer et al., 1999). However, *de novo* fungal fatty acid (FA) synthesis is observed only in colonized roots, not in extraradical hyphae or spores (Trépanier et al., 2005). In recent years, substantial progress has been made in elucidating this metabolism. Jiang et al. (2017), using techniques such as isotope labeling and split-root systems, demonstrated that FAs are the primary form of carbon supplied from plants to AM fungi. AM fungi primarily store carbon as triacylglycerol (TAG), and most FAs in AM fungi consist of 16:0 (palmitic acid) and 16:1 (palmitoleic acid) species within mycorrhized roots (Trépanier et al., 2005; Keymer et al., 2017). After they have penetrated plant roots, AM fungi can induce FA synthesis and transport to fulfill their nutritional requirements (Jiang et al., 2017; Luginbuehl et al., 2017). In plant root cells, lipid synthesis begins with acetyl-CoA (Shin and Ohlrogge, 2009). Sixteen-carbon FA chains are synthesized through the ketoacyl-ACP synthase (KAS) and fatty acid synthase (FAS) systems; they are released from FAS by acyl-ACP thioesterases (FatM). The saturated 16:0 FA is converted into 2-monoacylglycerol molecules by RAM2 within colonized plant cells and transported into the symbiotic space via the STR/STR2-mediated lipid export pathway (Jiang et al., 2017; Zhang et al., 2023). The lipid is subsequently taken up by unidentified lipid transporters in AM fungi. Recent studies have advanced understanding of how AM fungal infection activates lipid synthesis and transport in plant roots. Factors such as WRIs, CBX, ERM1, and PHRs play critical roles in facilitating lipid provision from plants to AM fungi (Jiang et al., 2018; Shi et al., 2021; Zhang et al., 2023).

As part of the symbiosis, the AM fungus penetrates plant roots and forms structures called hyphae and arbuscules within plant cells. AM symbiosis alters the levels of thousands of transcripts and metabolites in plants (Bravo et al., 2016; Jing et al., 2022; Shao et al., 2023). Additionally, plant hormones—critical regulators of growth and development and responses to environmental conditions—play key roles in modulating the interaction between plants and AM fungi (Liu and Harrison, 2020; Jing et al., 2022). Almost every known plant hormone contributes to AM formation and function, from early symbiosis pre-signaling to later stages, including the morphological changes in root cells required for fungal accommodation (Küster and Gutjahr, 2017; Pozo and López-Ráez, 2020). The germination of fungal spores, hyphal growth, and root colonization are stimulated by gibberellins (GAs) and auxins (Liao et al., 2018). In contrast, salicylates and ethylene exert negative effects during fungal invasion and root colonization (Blilou et al., 2000; Das et al., 2025). Notably, abscisic acid (ABA), a growth-inhibiting hormone, positively influences AM formation and function. ABA levels are greatly enhanced by AM fungi (Jing et al., 2022), suggesting that ABA plays a crucial role in AM symbiosis. There is evidence that ABA facilitates AM fungal growth and promotes arbuscule formation and hyphal elongation (Herrera-Medina et al., 2007; Charpentier et al., 2014; Liu et al., 2016). Numerous studies have demonstrated that ABA augments AM fungal symbiosis in crops (Charpentier et al., 2014; Miransari et al., 2014; Han et al., 2023), but the pathway through which AM fungi induce ABA to promote colonization remains unclear.

In this study, we investigated the role of elevated ABA levels in regulating lipid supplementation from apple plants to the AM fungus *Rhizophagus irregularis*. Increased ABA concentrations in apple roots promoted AM fungal growth. Inoculation with the AM fungus induced the expression of *MdNCED3.1* and *MdNCED3.2*, thereby increasing ABA biosynthesis in apple roots. ABA regulated FA synthesis and transport through the transcription factor MdABF2, enhancing FA supply and improving the symbiotic efficiency of AM. These results reveal a novel molecular mechanism by which ABA promotes AM symbiosis. The ABA signaling pathway positively regulates FA synthesis and transport, thereby increasing carbon supplementation to AM fungi and enhancing AM symbiosis.

## Results

### AM symbiosis induces an increase in ABA levels in apple roots

**Figure 1:**
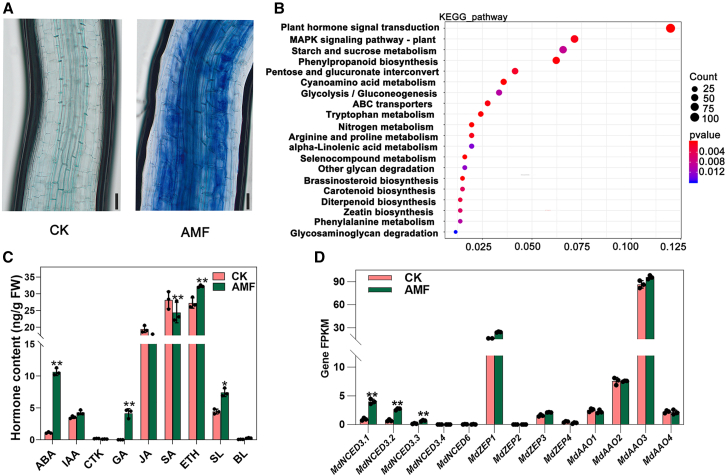
Roots of M26 (*M*. *pumila* Mill.) plants, uninoculated or inoculated with AM fungus, analyzed 60 days after inoculation via transcriptomic and metabolomic approaches. **(A)** Trypan blue staining of mycorrhizae in uninoculated (CK) and inoculated (AMF) M26 roots. Scale bars, 100 μm. **(B)** Kyoto Encyclopedia of Genes and Genomes-based enrichment analysis of DEGs in the root transcriptome. **(C)** Hormone content in M26 plants with and without inoculation. **(D)** Expression levels of ABA biosynthesis-related genes, measured by FPKM from RNA-seq, in uninoculated and inoculated M26 plants. Bars represent mean ± standard deviation (SD; *n* = 3, independent biological replicates). Mixed samples from three M26 plants were pooled as one replicate. Asterisks indicate significant differences as determined by one-way ANOVA (two-sided Student’s *t*-test; ∗∗*p* < 0.01, ∗*p* < 0.05).

As demonstrated in our previous report (Jing et al., 2022), AM symbiosis induces pronounced changes in phytohormone-related transcripts and metabolites in the roots of apple seedlings (Figures 1A–1C). According to liquid chromatography–tandem mass spectrometry (LC-MS/MS) analysis, among phytohormones, ABA content in roots increased nearly 10-fold after AM fungal inoculation compared with non-inoculated controls (Figure 1C). RNA sequencing (RNA-seq) showed that the expression of genes related to ABA biosynthesis (particularly *MdNCED)* also exhibited increasing trends, with significant upregulation after inoculation (Figure 1D). The transcript levels of *MdNCED3.1* and *MdNCED3*.*2* were considerably higher than those of other NCED family members, as determined by RNA-seq and quantitative polymerase chain reaction (qPCR) (Supplemental Figures 1 and 2). These results suggest that mycorrhizal symbiosis induces the transcription of ABA biosynthesis-related genes in apple roots.

### ABA facilitates colonization of AM fungi in apple roots

**Figure 2:**
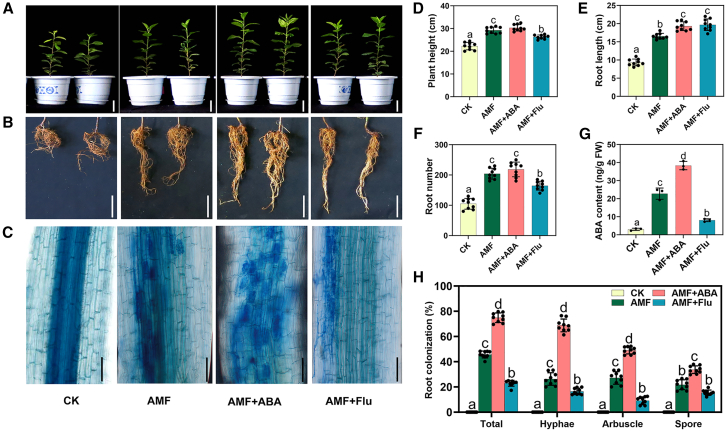
Exogenous ABA positively regulates AM symbiosis in M26 apple (*M*. *pumila* Mill.) plants. **(A–C)** Phenotypes of 45-day-old M26 plants under control (CK), AM-inoculated (AMF), inoculated plus ABA-treated (AMF+ABA), and inoculated plus ABA inhibitor-treated (AMF+Flu) conditions. **(A)** Plant height. Scale bars, 5 cm. **(B)** Root structure. Scale bars, 5 cm. **(A)** and **(B)** represent two replicates of the two plants. **(C)** Trypan blue staining of the fungus to visualize arbuscule morphology. Scale bars, 100 μm. **(D)** Plant height of M26 plants. **(E)** Root length of M26 plants. **(F)** Root number of M26 plants. **(G)** ABA content in M26 roots. Bars represent mean ± SD (*n* = 3, independent biological replicates). Mixed samples from three M26 plants were pooled as one replicate. **(H)** Quantification of mycorrhizal colonization levels. AMF, *R*. *irregularis*-inoculated plants without ABA treatment for 45 days; AMF+ABA, *R*. *irregularis*-inoculated plants treated with exogenous ABA (50 μM) for 15 days after 30 days of inoculation; AMF+Flu, *R*. *irregularis*-inoculated plants treated with exogenous ABA synthesis inhibitor fluridone (Flu, 50 μM) for 15 days after 30 days of inoculation; CK, uninoculated plants without ABA treatment for 45 days (control); FW, fresh weight. (**D–F**, and **H)** Bars represent mean ± SD (*n* = 9, independent biological replicates). (**D**–**H)** Different letters indicate significant differences according to one-way ANOVA followed by Duncan’s multiple range test (*p* < 0.05).

To determine whether ABA plays a role in the symbiosis between apple roots and AM fungi, we first examined the effects of exogenous ABA on *R*. *irregularis*. Thirty days after inoculation, various concentrations of exogenous ABA were applied to the root zone. Among these, 50 μM ABA most strongly promoted AM fungal development (Supplemental Figure 3). We then treated the apple plants with either 50 μM ABA or 50 μM fluridone (Flu, an ABA synthesis inhibitor) at 30 days after inoculation. After 15 days of treatment, there was a substantial increase in lateral roots among plants treated with ABA, whereas plants treated with Flu exhibited a significant reduction in lateral roots (Figures 2A, 2B, and 2D–2G). ABA treatment significantly enhanced AM symbiosis, whereas Flu treatment considerably suppressed AM symbiosis, particularly the formation of arbuscules and the growth of intercellular and intracellular hyphae (Figure 2C and 2H). These findings indicate that ABA is essential for AM symbiosis in apple.

The ABA synthesis genes *MdNCED3.1* and *MdNCED3.2* were both upregulated in apple roots after AM fungal inoculation (Figure 1D; Supplemental Figure 2). To determine whether *MdNCED3.1* and *MdNCED3.2* influence ABA synthesis, we generated independent *MdNCED3.1* and *MdNCED3.2* overexpression (OE) and RNA interference (RNAi) hairy root lines in *Malus hupehensis* Rhed, a triploid, typical apomictic species of *Malus*. In OE lines, *MdNCED3.1* and *MdNCED3.2* expression levels were significantly increased, whereas RNAi lines showed strong downregulation (Supplemental Figures 4D and 4E). To further evaluate the impacts of *MdNCED3.1* and *MdNCED3.2* on AM symbiosis, we divided transgenic seedlings into inoculated and non-inoculated groups. After 2 months, non-inoculated OE lines exhibited stunted shoot growth; RNAi lines showed no such inhibition but instead demonstrated increased root length and root number (Supplemental Figures 4A, and 4F–4H).

**Figure 3:**
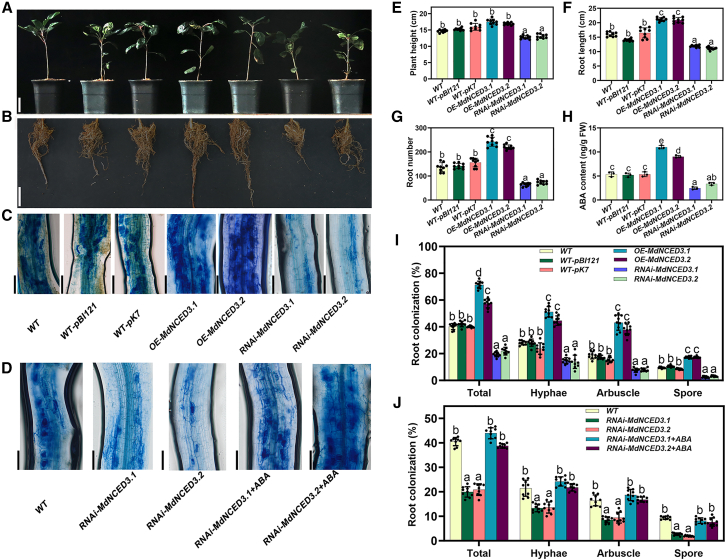
*MdNCED3.1* and *MdNCED3.2* positively regulate AM symbiosis in apple (*M*. *hupehensis* Rhed) seedlings. **(A–C)** Aboveground growth phenotypes **(A)**, root growth phenotypes **(B)**, and trypan blue staining of the fungus to visualize arbuscule morphology **(C)** in 60-day-old WT seedlings and hairy roots transformed with WT-pBI121 (empty vector), WT-pK7, OE-*MdNCED3.1*, OE-*MdNCED3.2*, RNAi-*MdNCED3.1*, or RNAi-*MdNCED3.2*. **(D)** Trypan blue staining of the fungus to visualize arbuscule morphology in WT, RNAi-*MdNCED3.1*, and RNAi-*MdNCED3.2* roots treated with ABA. **(E)** Plant height of apple seedlings carrying transgenic hairy roots after AM spore inoculation. **(F)** Root length of apple seedlings carrying transgenic hairy roots after AM inoculation. **(G)** Root number of apple seedlings carrying transgenic hairy roots after AM inoculation. **(H)** ABA content in roots of apple seedlings carrying transgenic hairy roots after AM inoculation. Bars represent mean ± SD (*n* = 3, independent biological replicates). Mixed samples from three apple seedlings carrying transgenic hairy roots were pooled as one replicate. **(I)** Quantification of mycorrhizal colonization levels in transgenic apple roots. **(J)** Quantification of mycorrhizal colonization levels in transgenic apple roots treated with ABA. RNAi-*MdNCED3.1*+ABA indicates *MdNCED3.1*-RNAi roots treated with exogenous ABA (50 μM) for 15 days. RNAi-*MdNCED3.2*+ABA indicates *MdNCED3.2*-RNAi roots treated with exogenous ABA (50 μM) for 15 days. **(A)** Scale bars, 5 cm. **(B)** Scale bars, 5 cm. **(C, D)** Scale bars, 100 μm. FW, fresh weight. Genetic modification of the apple seedlings was applied only to the roots. **(E**–**G**, **I and J)** Bars represent mean ± SD (*n* = 9, independent biological replicates). **(E**–**J)** Different letters indicate significant differences based on one-way ANOVA followed by Duncan’s multiple range test (*p* < 0.05).

Notably, OE of *MdNCED3.1* or *MdNCED3.2* in apple roots increased ABA content (Supplemental Figures 4I and 5) and significantly enhanced AM symbiosis after mycorrhizal inoculation (Figure 3C and 3I). In contrast, silencing *MdNCED3.1* or *MdNCED3.2* suppressed the ABA increase after fungal inoculation, reduced the infection rate by 50% compared with wild-type (WT) roots, and substantially impaired AM symbiosis, particularly arbuscule formation (Figures 3C and 3I). Application of exogenous ABA to RNAi*-MdNCED3.1* and RNAi-*MdNCED3.2* lines restored ABA content and partially rescued the arbuscular phenotype (Figures 3D and 3J; Supplemental Figure 6). These findings indicate that AM fungi induce *MdNCED3.1* and *MdNCED3.2* expression to promote endogenous ABA biosynthesis in apple roots, which is required for efficient colonization by AM fungi.

### The ABA signal promotes AM symbiosis via the transcription factor MdABF2

ABA mainly functions by regulating the expression of target genes, including transcription factors that mediate downstream signaling pathways (Yang et al., 2023). Analysis of our transcriptomic data for genes in the ABA signal transduction pathway revealed that the transcription factor *MdABF2* was significantly upregulated in roots inoculated with AM fungi relative to uninoculated roots (Supplemental Figure 7). The expression level of *MdABF2* was also significantly increased in roots treated exogenously with ABA and in the *MdNCED3.1*-OE and *MdNCED3.2*-OE lines (Supplemental Figure 8).

To clarify whether MdABF2 functions as an intermediary between ABA and AM symbiosis, we generated transgenic roots overexpressing and silencing *MdABF2* via hairy root *Agrobacterium* transformation. The OE seedlings exhibited significantly upregulated *MdABF2* expression, whereas the RNAi plants showed strong downregulation. Under non-inoculated conditions, *MdABF2*-overexpressing seedlings demonstrated enhanced growth compared with the WT (Supplemental Figure 9).

**Figure 4:**
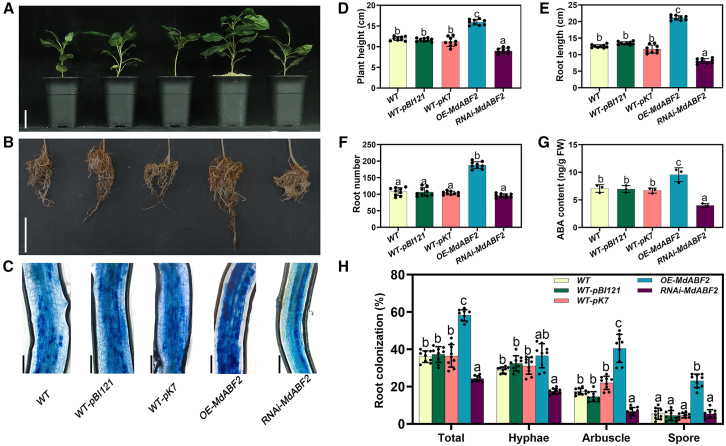
MdABF2 positively regulates AM symbiosis in apple (*M*. *hupehensis* Rhed) seedlings. **(A–C)** Aboveground growth phenotypes **(A)**, root growth phenotypes **(B)**, and trypan blue staining of the fungus to visualize arbuscule morphology **(C)** in 60-day-old WT seedlings, WT-pBI121 root transgenic lines, WT-pK7 transgenic lines, OE-*MdABF2* transgenic lines, and RNAi-*MdABF2* transgenic lines of apple seedlings. **(D)** Plant height of apple seedlings carrying transgenic hairy roots after AM spore inoculation. **(E)** Root length of apple seedlings carrying transgenic hairy roots after AM inoculation. **(F)** Root number of apple seedlings carrying transgenic hairy roots after AM inoculation. **(G)** ABA content in roots. Bars represent mean ± SD (*n* = 3, independent biological replicates). Mixed samples from three apple seedlings carrying transgenic hairy roots were pooled as one replicate. **(H)** Quantification of mycorrhizal colonization levels. **(A)** Scale bars, 5 cm. **(B)** Scale bars, 5 cm. **(C)** Scale bars, 100 μm. FW, fresh weight. Genetic modification of the apple seedlings was applied only to the roots. Bars represent mean ± SD. (**D**–**F**, and **H)** Bars represent mean ± SD (*n* = 9, independent biological replicates). (**D**–**H)** Different letters indicate significant differences determined by one-way ANOVA followed by Duncan’s multiple range test (*p* < 0.05).

We also introduced *pMdABF2::GUS* into apple roots; GUS staining revealed that *MdABF2* accumulates in arbuscule-containing cells (Supplemental Figure 10). Sixty days after AM inoculation, the *MdABF2*-OE lines displayed better root growth and aboveground development (Figures 4A, 4B, and 4D–4F) and greater AM symbiosis, particularly an increased number of arbuscules (Figure 4C and 4H), relative to the WT. In contrast, *MdABF2-*RNAi lines exhibited reduced arbuscule formation (Figure 4C and 4H). These results suggest that MdABF2 plays a key role in ABA-mediated progression of AM symbiosis.

### ABA signal influences FA levels during AM symbiosis

**Figure 5:**
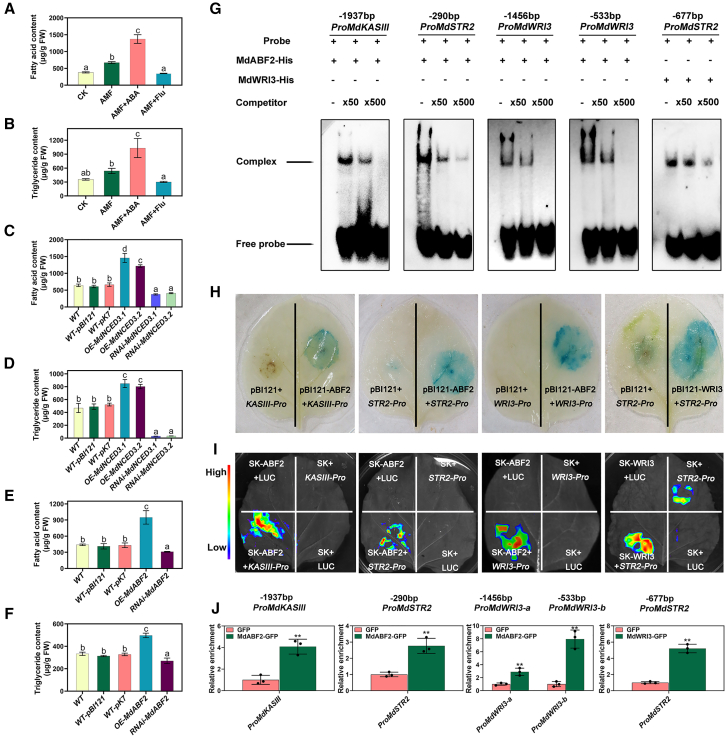
MdABF2 regulates the expression of *MdKASIII*, *MdSTR2*, and *MdWRI3*, and MdWRI3 regulates the expression of *MdSTR2*. **(A–F)** FA and triglyceride contents in apple roots after *R*. *irregularis* infection. **(A and B)** Non-transgenic M26 apple rootstock roots were analyzed for FA **(A)** and triglyceride **(B)** contents under CK, AMF, AMF+ABA, and AMF+Flu conditions. **(C, D)** Sixty-day-old apple (*M*. *hupehensis* Rhed) roots were analyzed for FA **(C)** and triglyceride **(D)** contents in WT and transgenic lines: WT-pBI121, WT-pK7, OE-*MdNCED3.1*, OE*-MdNCED3.2*, RNAi-*MdNCED3.1*, and RNAi*-MdNCED3.2*. **(E and F)** FA **(E)** and triglyceride **(F)** contents in transcription factor lines, including WT, WT-pBI121, WT-pK7, OE*-MdABF2*, and RNAi*-MdABF2*. AMF, mycorrhizal-inoculated plants without ABA treatment for 45 days; AMF+ABA, mycorrhizal-inoculated plants treated with exogenous ABA (50 μM) for 15 days after 30 days of inoculation; AMF+Flu, mycorrhizal-inoculated plants treated with exogenous Flu (50 μM ABA synthesis inhibitor fluridone) for 15 days after 30 days of inoculation; CK, non-mycorrhizal inoculated plants without ABA treatment for 45 days (control); FW, fresh weight. **(G)** EMSAs showing interactions of MdABF2 or MdWRI3 with 5′-biotin-labeled probes from the promoters of *MdKASIII*, *MdSTR2*, and *MdWRI3*. Competing unlabeled probes were used at two concentrations. **(H)** GUS staining demonstrating interactions of MdABF2 with the promoters of *MdKASIII*, *MdSTR2*, and *MdWRI3*, as well as the interaction of MdWRI3 with the *MdSTR2* promoter. Promoter-GUS fusions (*MdKASIII-Pro:GUS*, *MdWRI3-Pro:GUS*, or *MdSTR2-Pro:GUS*) were co-expressed with either the empty pBI121 vector or the pBI121 vector carrying the transcription factor *MdABF2*. *MdSTR2-Pro:GUS* was co-transformed with the *MdWRI3* OE vector (pBI121-Pro35S:MdWRI3) or an empty vector control (pBI121). **(I)** Dual-luciferase assays in tobacco leaves showing interactions of MdABF2 with the promoters of *MdKASIII*, *MdSTR2*, and *MdWRI3*, as well as the interaction of MdWRI3 with the *MdSTR2* promoter. Promoter-LUC fusions (*MdKASIII-Pro:LUC*, *MdWRI3-Pro:LUC*, *MdSTR2-Pro:LUC*) and empty vector (pGreenII 0800-LUC) were co-transformed with either the MdABF2 OE vector (*Pro35S:MdABF2*) or an empty vector control (pGreenII 62-SK). *MdSTR2-Pro:LUC* and the empty vector (pGreenII 0800-LUC) were also co-transformed with either the MdWRI3 OE vector (*Pro35S:MdWRI3*) or an empty vector control (pGreenII 62-SK). **(J)** ChIP–qPCR showing MdABF2 binding to the promoters of *MdKASIII*, *MdSTR2*, and *MdWRI3 in vivo*, as well as MdWRI3 binding to the *MdSTR2* promoter *in vivo*. Bars represent mean ± SD (*n* = 3, independent biological replicates). **(A and B)** Mixed samples from three M26 plants were pooled as one replicate. (**C**–**F,** and **J)** Mixed samples from three apple seedlings carrying transgenic hairy roots were pooled as one replicate. Different letters indicate significant differences as determined by one-way ANOVA followed by Duncan’s multiple range test (*p* < 0.05).

FAs are the primary form of carbon supplied from plant roots to AM fungi, and their levels are crucial for the development of symbiosis (Jiang et al., 2017; Rich et al., 2017; Ivanov and Harrison, 2024). We measured the FA and TAG contents in the roots of WT M26 plants treated with exogenous ABA and Flu, as well as in various transgenic hairy root lines (Figures 5A and 5B; Supplemental Figure 11). The results indicated that—within an appropriate concentration range—ABA increases FA and TAG contents in mycorrhizal roots. Disruption of ABA biosynthesis or signaling reduced FA and TAG contents. Furthermore, changes in *MdNCED* expression levels were linked to FA and TAG contents in mycorrhizal roots. Alterations in *MdABF2* expression levels were also correlated with changes in FA and TAG contents in mycorrhizal roots (Figures 5C–5F; Supplemental Figure 12).

Next, we examined the relative expression of genes involved in FA synthesis and transport in apple roots under symbiotic and non-symbiotic conditions. The results indicated that elevated ABA levels increase the relative expression levels of FA synthesis- and transport-related genes (*MdKASIII/MdSTR2*) in mycorrhizal roots. Disruption of ABA biosynthesis or signaling reduced the relative expression of these genes. Changes in *MdNCED* expression levels were linked to the relative expression of *MdKASIII* and *MdSTR2* in mycorrhizal roots. Similarly, alterations in *MdABF2* expression levels were correlated with changes in the relative expression of *MdKASIII* and *MdSTR2* in mycorrhizal roots. We also observed that *MdPT4* expression was strongly induced by AM fungi (Supplemental Figures 13 and 14).

### The transcription factor MdABF2 regulates the expression of FA synthesis genes in apple roots

Subsequently, we analyzed differentially expressed genes (DEGs) involved in lipid metabolism using two transcriptome datasets: one comparing roots of mycorrhizal and non-mycorrhizal plants sampled 60 days after inoculation, and the other from mycorrhizal roots of transgenic lines overexpressing or silencing *MdABF2* sampled at the same time point. DEGs shared between the two datasets included *MdKASI*, *MdKASI-1*, *MdKASIII*, *MdRAM2*, *MdRAM2-1*, *MdSTR2*, and *MdWRI3*. These genes were significantly upregulated after AM fungal inoculation, and this upregulation required the *MdABF2* transcription factor (Supplemental Figure 15; Supplemental Data 2). The promoter regions of these seven genes each contained at least one ABRE element motif ((C/T)ACGTn), a potential binding site for ABF (Supplemental Figure 16A). Additionally, the *MdSTR2* promoter region contained an AW-box motif (CGnnn(n)_4_CnAnG), a potential binding site for WRI3 (Supplemental Figure 16C).

Preliminary yeast one-hybrid (Y1H) assays showed that MdABF2 can bind to the promoters of the FA synthesis genes *MdKASI*, *MdKASI-1*, *MdKASIII*, *MdRAM2*, and *MdRAM2-1*; the transport protein gene *MdSTR2*; and the transcription factor *MdWRI3 in vitro* (Supplemental Figure 16B). Similarly, MdWRI3 demonstrated binding to the promoter of *MdSTR2 in vitro* (Supplemental Figure 16D). The genes *MdKASIII*, *MdWRI3*, and *MdSTR2* exhibited higher transcript abundance in apple roots, with pronounced upregulation after AM inoculation (Supplemental Figures 15 and 17).

To further explore whether MdABF2 regulates the expression of genes involved in FA synthesis, transport, and transcriptional regulation, or whether MdWRI3 regulates *MdSTR2* expression through physical interaction with its promoter, we performed electrophoretic mobility shift assay (EMSA), β-glucuronidase (GUS) reporter analysis, dual-luciferase assay, and chromatin immunoprecipitation qPCR (ChIP–qPCR), as described below.

*In vitro*, EMSAs showed that promoter fragments of *MdKASIII*, *MdWRI3*, and *MdSTR2* containing ABRE motifs were bound by recombinant MdABF2-His, resulting in a mobility shift. A similar shift was observed for the interaction between MdWRI3-His and the *MdSTR2* promoter fragment (Figure 5G). *In vivo*, GUS enzyme activity assays demonstrated that co-expression of *MdSTR2-Pro:GUS* with *35S:MdWRI3*, and of either *MdWRI3-*, *MdKASIII-*, or *MdSTR2-Pro:GUS* with *35S:MdABF2*, significantly increased GUS staining in tobacco leaves (Figure 5H). The dual-luciferase assay confirmed that co-expression of the transcription factor *35S:MdABF2* significantly enhanced luciferase activity when the *LUC* gene was driven by the promoter of either *MdWRI3*, *MdKASIII*, or *MdSTR2* in tobacco leaves, relative to other combinations. Similarly, co-expression of the transcription factor *35S:MdWRI3* with *MdSTR2-Pro:LUC* significantly increased luciferase activity compared with other combinations (Figure 5I; Supplemental Figure 18). The ChIP–qPCR analysis demonstrated that MdABF2 binds to the promoters of *MdKASIII*, *MdSTR2*, and *MdWRI3*; additionally, MdWRI3 binds to the promoter of *MdSTR2* (Figure 5J). Overall, these results indicate that MdABF2 directly binds to the promoter sequences of *MdKASIII*, *MdWRI3*, and *MdSTR2*, and that MdWRI3 directly binds to the *MdSTR2* promoter, thus promoting transcription of genes involved in FA synthesis and transport.

### Synthesis and transport of FAs support AM symbiosis in apple roots

**Figure 6:**
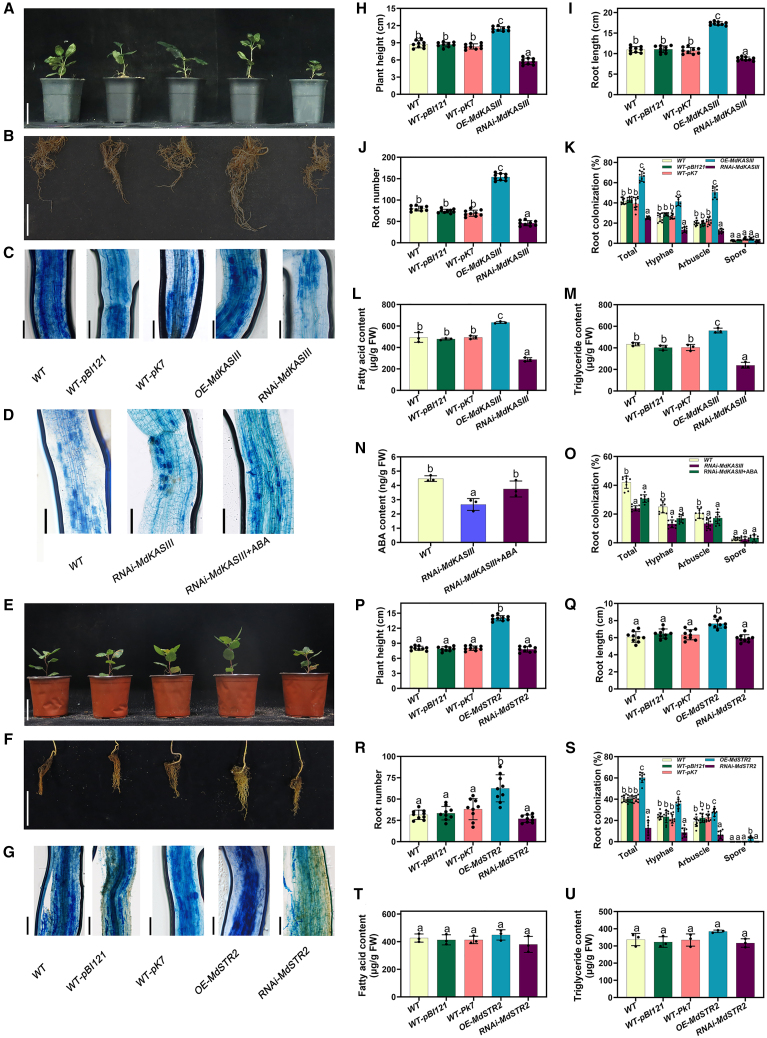
*MdKASIII* and *MdSTR2* positively regulate AM symbiosis in apple (*M*. *hupehensis* Rhed) seedlings. Aboveground growth phenotypes **(A)**, root growth phenotypes **(B)**, and trypan blue staining of the fungus to visualize arbuscule morphology **(C)** in 60-day-old WT, WT-pBI121, WT-pK7, OE-*MdKASIII*, and RNAi-*MdKASIII* transgenic hairy root apple lines. **(D)** Trypan blue staining of the fungus to visualize arbuscule morphology in WT and RNAi-*MdKASIII* roots treated with ABA. Aboveground growth phenotypes **(E)**, root growth phenotypes **(F)**, and trypan blue staining of the fungus to visualize arbuscule morphology **(G)** in 60-day-old WT, WT-pBI121, WT-pK7, OE-*MdSTR2* and RNAi-*MdSTR2* transgenic hairy root apple lines. (**H**–**O)** Statistical data related to phenotypes of the *MdKASIII* transgenic hairy root apple lines. **(H)** Plant height of apple seedlings carrying transgenic hairy roots after AM spore inoculation. **(I)** Root length of apple seedlings carrying transgenic hairy roots after AM inoculation. **(J)** Root number of apple seedlings carrying transgenic hairy roots after AM inoculation. **(K)** Quantification of mycorrhizal colonization levels. FA **(L)** and triglyceride (**M)** contents in transgenic apple roots. **(N)** ABA content in transgenic apple roots treated with ABA. **(O)** Quantification of mycorrhizal colonization levels in transgenic apple roots treated with ABA. RNAi-*MdKASIII*+ABA indicates *MdKASIII*-RNAi roots treated with exogenous ABA (50 μM) for 15 days. Genetic modification of the apple seedlings was applied only to the roots. (**H–K,** and **O)** Bars represent mean ± SD (*n* = 9, independent biological replicates). (**L**–**N**) Bars represent mean ± SD (*n* = 3, independent biological replicates). Mixed samples from three apple seedlings carrying transgenic hairy roots were pooled as one replicate. (**H**–**O**) Different letters indicate significant differences based on one-way ANOVA followed by Duncan’s multiple range test (*p* < 0.05). (**P–U**) Statistical data related to phenotypes of the *MdSTR2* transgenic hairy root apple lines. **(P)** Plant height of apple seedlings carrying transgenic hairy roots. **(Q)** Root length of apple seedlings carrying transgenic hairy roots. **(R)** Root number of apple seedlings carrying transgenic hairy roots. **(S)** Quantification of mycorrhizal colonization levels. FA **(T)** and triglyceride **(U)** contents in transgenic apple roots. **(A)** Scale bars, 5 cm. **(B)** Scale bars, 5 cm. **(C**, **D)** Scale bars, 100 μm. **(E)** Scale bars, 5 cm. **(F)** Scale bars, 5 cm. **(G)** Scale bars, 100 μm. FW, fresh weight. Genetic modification of the apple seedlings was applied only to the roots. (**P–S)** Bars represent mean ± SD (*n* = 9, independent biological replicates). **(T** and **U)** Bars represent mean ± SD (*n* = 3, independent biological replicates). Mixed samples from three apple seedlings carrying transgenic hairy roots were pooled as one replicate. (**P–U)** Different letters indicate significant differences based on one-way ANOVA followed by Duncan’s multiple range test (*p* < 0.05).

To determine whether *MdKASIII* influences FA synthesis and AM symbiosis in apple roots, we generated transgenic hairy roots either overexpressing or silencing *MdKASIII*. The OE seedlings showed significantly upregulated *MdKASIII* expression, whereas the RNAi seedlings exhibited strong downregulation. Under non-inoculated conditions, seedlings overexpressing *MdKASIII* displayed improved growth compared with the WT; seedlings with *MdKASIII* silencing exhibited reduced growth relative to the WT (Supplemental Figure 19). Overexpression of *MdKASIII* increased FA and TAG contents and enhanced AM symbiosis in apple roots, particularly promoting arbuscule formation. Silencing of *MdKASIII* decreased FA and TAG contents and reduced AM colonization. Application of exogenous ABA to the RNAi*-MdKASIII* lines partially restored the arbuscular phenotype (Figure 6; Supplemental Figure 19). These results indicate that KASIII affects AM symbiosis by modulating FA accumulation.

Bimolecular fluorescence complementation assays demonstrated that MdSTR2 can form homodimers in apple (Supplemental Figure 21). In the absence of AM inoculation, neither OE nor silencing of *MdSTR2* significantly influenced plant growth and development. However, under AM-inoculated conditions, *MdSTR2* OE enhanced aboveground plant growth (Figure 6; Supplemental Figure 22). Similarly, OE of the transporter *MdSTR2* promoted AM symbiosis in apple roots, particularly increasing arbuscule formation (Figures 6G and 6S). OE of *MdSTR2* did not result in a significant difference in total FA or TAG contents in the overexpressing roots (Supplemental Figures 22H and 22I; Figures 6T and 6U). These findings suggest that MdSTR2 influences AM symbiosis, likely by modifying FA transport rather than synthesis. OE of *MdSTR2* increased expression levels of *MdSTR*-1 and *MdSTR*-2 in apple roots (Supplemental Figure 24). Furthermore, we observed that *MdPT4* expression was strongly induced by AM fungi (Supplemental Figures 20 and 23). Collectively, these results support the conclusion that the transcription factor MdABF2 regulates AM symbiosis by controlling the expression of genes involved in FA synthesis and transport to AM hyphae in apple roots.

## Discussion

AM fungi are beneficial symbionts that enhance nutrient uptake and stress resistance in their host plants (Zhou et al., 2015; Bernaola et al., 2018). However, the natural colonization rate of AM fungi is low under production conditions (Vallino et al., 2009; Lumini et al., 2011). Therefore, it is important to improve the symbiosis rate of AM fungi in plant roots. Although low-phosphorus and low-nitrogen treatments have been shown to increase infection rates, these approaches are difficult to implement in modern agricultural systems (Breuillin et al., 2010; Balzergue et al., 2011; Kobae et al., 2016). Thus, efforts to explore the infection mechanisms of AM fungi and enhance colonization by modifying plant traits hold great potential.

### Increased ABA synthesis is necessary for the formation of AM symbiosis

ABA plays an indispensable role in the establishment of symbiosis between apple and the fungus *R.* *irregularis*. The increase in ABA during AM fungal infection is attributed to endogenous ABA biosynthesis in the apple plant (Figures 1D and 3; Supplemental Figure 4). Specifically, the AM fungus either directly or indirectly activates the plant’s ABA biosynthesis pathway by inducing genes such as *NCED*, a key regulator of ABA production. Factors responsible for the upregulation of *NCED* remain unclear, but three potential pathways can be considered. First, AM fungi release numerous effector molecules that reprogram plant cells (Betz et al., 2024). The influence of effectors on *NCED* expression is plausible—Li et al. (2020) demonstrated that effector proteins can disrupt the localization of HbNCED5 and inhibit ABA biosynthesis. Second, ABA synthesis is influenced by other plant hormones (Cutler et al., 2010; Nakashima and Yamaguchi-Shinozaki, 2013). During AM symbiosis, the levels of strigolactones (SLs), salicylic acid (SA), jasmonic acid (JA), and ethylene (Eth) are also altered, although to a lesser extent than ABA (Figure 1C). Nevertheless, even minor changes in these endogenous hormones may modulate *NCED* expression. Studies have shown that Eth, SA, and JA can directly or indirectly influence the expression of genes encoding key enzymes in ABA biosynthesis, such as *NCED* (Peleg and Blumwald, 2011; Zhang et al., 2016). Third, the increase in *NCED* expression may result from the plant’s efforts to mitigate excess reactive oxygen species (ROS). AM fungal invasion generates substantial ROS in plants (Salzer et al., 1999; Espinosa et al., 2014). To reduce oxidative damage, the plant may upregulate *NCED* expression, thus increasing ABA levels and activating ABA signaling. In summary, the upregulation of *NCED* during AM fungal symbiosis may represent an important regulatory mechanism by which plants respond to stress and initiate or maintain symbiotic relationships.

Intriguingly, the elevated ABA levels during AM symbiosis did not inhibit plant growth or development (Figures 1 and 2). This phenomenon is likely related to suppression of the ABA signaling pathway (Santner and Estelle, 2009). Although ABA content substantially increased during AM fungal colonization, downstream ABA signal transduction genes, such as *PYL* and *SnRK*, showed no significant changes before and after inoculation (Supplemental Data 1). Furthermore, plants often maintain hormonal homeostasis during growth and development (Depuydt and Hardtke, 2011; Vanstraelen and Benková, 2012). Although ABA levels substantially increased, the concentrations of growth-promoting hormones (e.g., gibberellins, SLs, and brassinolide) also significantly rose during AM fungal infection (Figure 1C).

### ABA regulates the synthesis and transport of FAs to supply carbon for AM fungal cells in roots

During AM symbiosis, FA serves as a critical nutrient source for AM fungi. Genes involved in FA biosynthesis, such as *KAS*, *FatM*, and *RAM*, are induced by AM fungi, and OE of these genes promotes mycorrhizal symbiosis (Jiang et al., 2017). Our study demonstrated that ABA modulates AM symbiosis by regulating FA synthesis and transport (Figures 4, 5, and 6). However, the partial recovery of arbuscule formation observed in RNAi*-MdKASIII* lines after exogenous ABA application (Figure 6) suggests the existence of additional pathways through which ABA promotes AM symbiosis.

The regulatory hierarchy centered on ABF, which controls the direct FA metabolism pathway, appears to be conserved across various plant species (Yeap et al., 2017; Li et al., 2020). Numerous studies have also shown a close relationship between ABA and FA metabolism. In oil palm, EgABI5 transcriptionally activates *EgDGAT1*, promoting oil biosynthesis during fruit development (Yeap et al., 2017). In our study, ABA, through the transcription factor ABF2, directly or indirectly regulated genes involved in FA synthesis (*KASI* and *KASIII*) and transport (*STR2*), thereby influencing FA supply and promoting AM symbiosis (Figures 4, 5, and 6). These findings indicate that ABF2 is critical for AM symbiosis.

In *Medicago truncatula*, the AP2-domain transcription factor WRI5a has been identified as the primary regulator of lipid biosynthesis and transfer during AM symbiosis; WRI5a can regulate *STR* expression (Jiang et al., 2018). We found that ABF2 activates the expression of *MdWRI3*, a homolog of WRI5a. ABF2 can directly regulate STR2 expression or indirectly modulate STR2 through WRI3. STR controls lipid flux from the plant to the AM fungus (Zhang et al., 2010; Gutjahr et al., 2012; Jiang et al., 2017).

It is noteworthy that plants possess two regulatory pathways to transfer FA to AM fungi. In our study, multiple genes associated with FA synthesis, including *RAM2*, *RAM2-1*, *KAS1*, *KAS1-1*, *KASI*, and *KASIII*, were significantly upregulated (Supplemental Figure 15), leading to a pronounced increase in FA content, particularly TAG (Figure 6). In contrast, only one gene associated with FA transfer, *STR2*, was upregulated (Supplemental Figure 15). Given our findings that ABF2 regulates *STR2 expression* both directly and indirectly (Figure 5), we propose that AM fungi have a higher demand for FA compared with other carbon compounds in apple roots. The substantial increase in FA content in mycorrhizal roots likely requires activation of transfer pathways to meet the nutritional demands of AM fungi.

**Figure 7:**
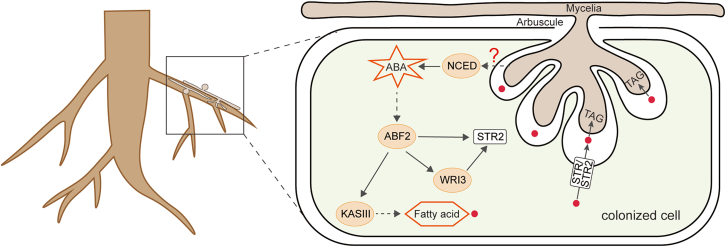
Proposed pathway through which ABA regulates the supplementation of FAs to AM fungi during symbiosis in root cells. Infection by AM fungi induces ABA synthesis through the upregulation of *MdNCED3.1* and *MdNCED3.2* in roots via an unknown pathway. Elevated ABA levels subsequently increase the expression of genes involved in FA synthesis (e.g., *MdKASIII*) and FA transport (*MdSTR2*) through the MdABF2-mediated signaling pathway. This process results in a carbon supply from plant roots that supports fungal growth and promotes AM symbiosis.

Overall, this research represents significant progress in understanding the molecular mechanisms by which ABA promotes AM fungal colonization during the complex interaction between plants and fungi. The results demonstrated that ABA is essential for efficient formation of AM symbiosis in apple roots. AM fungal infection induced ABA biosynthesis, accompanied by upregulated expression of *MdNCED3.1* and *MdNCED3.2* in the roots through an unknown pathway. The increased ABA levels upregulated the expression of genes involved in both FA synthesis (e.g., *MdKASIII*) and FA transport (*MdSTR2*) via the MdABF2 signaling pathway. Elevated FA levels in plant roots provide a primary carbon source for fungal growth, thereby promoting AM symbiosis (Figure 7). Other studies have also shown that ABA biosynthesis considerably increases during AM symbiosis. One potential strategy to improve AM symbiosis in crops, with the goal of enhancing yield and fruit quality, may involve increasing ABA levels in roots. Further investigation of the specific mechanisms by which AM symbiosis influences plant metabolism and development is both necessary and feasible to support the utilization of AM fungi for improved crop productivity and quality.

## Materials and Methods

### Plant and fungal materials

This experiment was conducted in Shaanxi Province at Northwest A&F University. Plants were maintained in a greenhouse in plastic pots filled with sand sterilized by steaming at 121°C for 2 h. The AM fungus *R.* *irregularis* (line number: BGC BJ09) was stored in a refrigerator at 4°C. Spores were isolated and counted as previously described (Gerdemann and Nicolson, 1963). The inoculant contained approximately 60 spores per gram.

Apple rootstock cultivar M26 (*M.* *pumila* Mill.) plants and *M*. *hupehensis* Rhed seedlings were used in these experiments. M26 was used to evaluate the effect of exogenous ABA treatment on AM symbiosis and for omics sequencing. *M*. *hupehensis* Rhed, a triploid and typical apomictic species of *Malus*, was selected for its susceptibility to hairy root transformation by *Agrobacterium rhizogenes*.

### Experimental treatments

For all experiments, the roots of apple plants or seedlings were inoculated with 20 g of AM fungal inoculant, whereas the control group received 20 g of sterilized AM fungal spores. After treatment, the plants were placed in a greenhouse maintained at a relative humidity of 55% to 75% and temperatures ranging from 12°C to 30°C. The plants were watered regularly; all groups received weekly irrigation with 100 mL of half-strength, low-phosphorus Hoagland’s nutrient solution.

For transcriptome sequencing and metabolic analyses, 200 saplings of apple rootstock M26 were used, with 100 plants assigned to the treatment group and 100 to the control group. The plants, approximately 10 cm in height, were grown in plastic pots (21 cm × 15 cm). Sixty days after AM fungal inoculation, measurements were recorded and root samples were collected.

Apple rootstock M26 was also used for exogenous ABA treatment and planted in plastic pots (14 cm × 12 cm). Plants approximately 10 cm in height were selected. One-quarter of the plants remained uninoculated and untreated for 45 days as the control group. Another quarter of the plants were inoculated with AM fungal spores and grown for 45 days. A third group received AM fungal inoculation and, after 30 days, was treated with exogenous ABA (50 μM) for an additional 15 days. The fourth group was inoculated with AM fungi and treated with exogenous fluridone (Flu, 50 μM) 30 days after inoculation, followed by 15 days of growth before sampling. Plant indices were measured, and samples were collected 45 days after AM fungal inoculation.

Apple seedlings (*M.* *hupehensis* Rhed), used to generate *MdNCED3.1* and *MdNCED3.2* transgenic hairy roots, were planted in plastic pots (8.8 cm × 9 cm). One portion of the seedlings was inoculated with AM fungal spores, whereas the other remained uninoculated. Seedlings in these groups were not transformed and served as WT controls. Additional seedlings were co-cultured with *A*. *rhizogenes* harboring either the pBI121 empty vector, pK7 empty vector, *pBI121-MdNCED3.1*, *pBI121-MdNCED3.2*, *pK7-MdNCED3.1*, or *pK7-MdNCED3.2*. Uninoculated transgenic lines were divided into seven groups as controls. Inoculated transgenic lines were assigned to experimental groups accordingly. After hairy root transformation with *A. rhizogenes*, the plants were cultured for 60 days; they were then subjected to phenotypic assessment and sample collection.

The *MdABF2*, *MdKASIII*, and *MdSTR2* transgenic materials were developed and treated in the same manner as the *MdNCED3.1* and *MdNCED3.2* roots.

### Mycorrhizal colonization and morphological index assays

The method used to observe mycorrhizal colonization followed the protocol of He et al. (2016), with minor modifications. The roots were cleaned, soaked in 10% KOH until transparent and softened, stained with 0.05% trypan blue/wheat germ agglutinin-Alexa Fluor 633, and decolorized three times with a 1:1 acetic acid–glycerin solution before microscopic examination. At the end of the 60-day co-culture period, plant height and root length were measured using a steel ruler, and the number of lateral roots was manually counted.

### Transcriptome analysis of apple roots

Root tips were used to extract RNA in accordance with the Plant Total RNA Isolation Kit (FOREGENE) protocol. Primer sequences are provided in Supplemental Data 3. A high-quality cDNA library was constructed, and transcriptome sequencing was performed on an Illumina HiSeq platform (Illumina). Raw data in FASTQ format (original reads) were processed using an internal Perl script. After data processing, high-quality reads were obtained and mapped to the reference genome (https://www.rosaceae.org/species/malus/all). The FPKM value for each gene was calculated. Principal component analysis was conducted to evaluate relationships among the samples. DEGs were identified based on *p*-values < 0.05 and fold change values ≥ 2.0. Functional annotation of DEGs was performed using the Kyoto Encyclopedia of Genes and Genomes (KEGG) and MapMan databases.

### Hormone analysis in apple roots

Hormone metabolomics were utilized to identify differential hormone levels via ultra-performance LC-MS/MS analysis. Briefly, 0.05 g of root tip samples were mixed with 10 μL of a mixed internal standard solution (10 ng/mL) and 1 mL of methanol, water, and formic acid (15:4:1, v/v/v), then vortexed thoroughly. A Thermo Vanquish UPLC system equipped with a Waters ACQUITY UPLC HSS T3 C18 column (1.8 μm, 100 mm × 2.1 mm inner diameter) was used. The program was set with a flow rate of 0.35 mL/min, column temperature of 40°C, and injection volume of 4 μL per run. Solvents A and B consisted of water with 0.04% acetic acid and acetonitrile with 0.04% acetic acid, respectively. For MS analysis, the electrospray ionization temperature was set at 550°C, and the curtain gas pressure was 35 psi. Principal component analysis was conducted to assess relationships among samples. Differential hormone metabolite levels were identified based on *p*-values < 0.05 and fold change values ≥ 2.0.

### Hairy root *Agrobacterium* transformation and mycorrhizal colonization

To construct OE and RNAi vectors, the coding sequences of *MdNCED3.1*, *MdNCED3.2*, *MdABF2*, *MdKASIII*, and *MdSTR2* were cloned into a pBI121 vector containing the 35S promoter. Additionally, specific truncated sequences (200–300 bp within the coding region) were inserted into a pK7GWIWG2 vector. These fusion vectors, along with pBI121-GFP and pK7GWIWG2, were introduced into *A.* *rhizogenes* K599 (Weidi Biotechnology, Shanghai, China).

Transgenic seedlings were generated using *A.* *rhizogenes* to induce hairy roots, following a method similar to that described by Boisson-Dernier et al. (2001). Sixty days after co-culture with *A. rhizogenes*, the roots were inoculated with *R. irregularis* spores according to the protocol of Jiang et al. (2017).

For apple root transformation, apple seeds were germinated on petri dishes lined with wet filter paper. Once the seedlings developed three cotyledons, *A. rhizogenes* strain Arqua-1 carrying the desired vector was used to transform and generate hairy roots. Each vector included a green fluorescent protein (GFP) reporter gene.

After 3–4 weeks, untransformed roots were excised based on GFP fluorescence observed under a somatic fluorescence microscope (Zeiss). Apple seedlings with transformed roots, along with control plants (untransformed WT or those harboring empty vectors), were transplanted into sand, inoculated with approximately 60 *R. irregularis* spores per plant, and cultivated in the greenhouse.

### Quantification of FAs

The triglyceride content in roots was extracted following the method of Li et al. (2017). The FA content in roots was measured following the protocol of Shi et al. (2018). FA and triglyceride analyses were performed using an Agilent 7890B gas chromatograph equipped with an HP-88 column (RESTEK; 0.25 mm inner diameter, 100 m length, and 0.250 μm film thickness; Agilent).

FA quantification was carried out via the external standard method. An FA methyl ester mixed standard (GLC NESTLE 37 MIX, Solarbio) was used to generate the FA methyl ester standard curve.

### Quantification of ABA

ABA was extracted using the ethyl acetate method, and its content was determined by high-performance liquid chromatography (Müller and Munné-Bosch, 2011). High-performance LC-MS (QTRAP5500, AB SCIEX) was performed using a packed column (150 mm × 4.6 mm, 5 μm; InsertContinustTM AQ-C18, Shimadzu, Japan). Mobile phase A consisted of 0.1% formic acid (85 178, Thermo Fisher Scientific), and mobile phase B was methanol (67 561, Thermo Fisher Scientific).

### Gene expression analysis

Roots were used for RNA extraction with the Plant Total RNA Isolation Kit (FOREGENE). All primers are listed in Supplemental Data 3, and gene IDs are provided in Supplemental Data 4. For quantitative reverse transcription–PCR reactions, cDNA was synthesized using PrimeScript RT reagent (Takara, http://www.takarabiomed.com.cn). The 2× Fast qPCR Master Mixture (DiNing, https://di-ning.com.cn/) was used for cDNA analysis on the iQ5 Multicolor Real-Time PCR Detection System (Bio-Rad). Data were analyzed using the iQ5 2.0 standard optical system analysis software and the 2^−ΔΔCT^ method (Livak and Schmittgen 2001).

### Phylogenetic analysis

To identify potential *NCED* genes in the apple genome, known *NCED* genes of *Arabidopsis* were used as queries for TBLASTN searches against the apple genome. Multiple sequence alignments of the amino acid sequences were constructed using MEGA7 with 1,000 bootstrap replicates.

### Y1H assay

The Matchmaker Gold Y1H system was used for Y1H assays in *Saccharomyces cerevisiae* (Takara, https://www.takarabiomed.com.cn). Reporter strains were generated by inserting the promoters of *MdKASI*, *MdKASIII*, *MdRAM2*, and *MdSTR2* into pAbAi vectors, which were then transformed into Y1H Gold. The minimal concentration of Aureobasidin A required to inhibit growth was determined for each strain on SD/-Ura plates. The full-length coding sequence of *MdABF2* was cloned into the pGADT7-Rec vector. The *MdABF2*-pGADT7-Rec construct was transformed into each promoter-reporter strain according to the manufacturer’s protocol. Transformed yeast cells were plated on SD/-Leu/ABA medium and incubated at 30°C for 3 days.

### GUS staining

The promoters of *MdKASIII*, *MdWRI3*, and *MdSTR2* were cloned into the pC0390-GUS vector, whereas the open reading frame (ORF) sequences of *MdABF2* and *MdWRI3* were inserted into the pBI121OE vector (OE vector). The fusion constructs were transformed into *Agrobacterium tumefaciens* GV3101 (pSoup-p19) (Weidi Biotechnology).

The construct 35S:MdABF2 was co-infiltrated into tobacco leaves along with either *proMdKASIII-GUS*, *proMdSTR2-GUS*, or *proMdWRI3-GUS*. Similarly, 35S:MdWRI3 was co-infiltrated with *proMdSTR2-GUS*. Histochemical staining was performed to detect GUS activity in the transformants (Zhu et al., 2023; Gao et al., 2024). Each treatment included three biological replicates. Primers used for these experiments are listed in Supplemental Data 3.

### Luciferase complementation assay

The ORF sequences of *MdABF2* and *MdWRI3* were cloned into the pGreenII 62-SK vector. The promoters of *MdKASIII*, *MdSTR2*, and *MdWRI3* were cloned into the pGreenII 0800-LUC vector. The fusion constructs were transformed into *A*. *tumefaciens* GV3101 (pSoup-p19) (Weidi Biotechnology). The *A. tumefaciens* strains were co-infiltrated into tobacco leaves in various pairings. Bioluminescence intensity was measured using a live plant imaging system (PlantView100, Guangzhou Biolight Biotechnology Co.).

### EMSA

Purified MdABF2-His and MdWRI3-His recombinant proteins were produced in accordance with the protocol for Ni-NTA binding resin (7 Sea Biotech). Promoter probes were amplified using primers with biotin-labeled oligonucleotides (Invitrogen) or unlabeled oligonucleotides (used as competitors), as listed in Supplemental Data 3. EMSA reactions were performed as described by Zheng et al. (2018). Each experiment was independently repeated three times.

### ChIP–qPCR assay

For the ChIP–qPCR assay, the coding sequences of *MdABF2* and *MdWRI3* (excluding stop codons) were inserted into the pBI121 vector containing a C-terminal GFP tag. The recombinant constructs were transformed into apple, with 35S:GFP serving as a control, to generate GFP-tagged transgenic material for use in ChIP–qPCR analysis.

### Bimolecular fluorescence complementation assays

To investigate protein–protein interactions, MdSTR2 was fused to the N-terminal fragment of YFP, as well as the C-terminal fragment of YFP. The fusion constructs were transformed into *A.* *tumefaciens* GV3101 (pSoup-p19) (Weidi Biotechnology) and co-infiltrated into tobacco leaves. After infiltration, the tobacco plants were kept in darkness for 12 h, followed by 48 h of light exposure to allow protein expression. Fluorescence signals were detected using a confocal microscope (FV3000, Olympus).

### Statistical analysis

Data were analyzed using GraphPad Prism 10.1.2 and IBM SPSS Statistics 26. Significant differences were determined via one-way analysis of variance (ANOVA) followed by a two-sided Student’s *t*-test or Duncan’s multiple range test (*p* < 0.05).

## Data availability

All data generated or analyzed in this study are included in the published article and its supplemental information files. Raw data are available from the corresponding author upon reasonable request. The data presented in this study have been deposited in the NCBI repository under accession number PRJNA889983.

## Funding

This work was supported by the 10.13039/501100012166National Key Research and Development Program of China (2023YFD2301000), the Shaanxi Science and Technology Innovation Team Project (2022TD-12), the Young Elite Scientists Sponsorship Program by CAST (2023QNRC001), the 10.13039/501100017550Shaanxi Association for Science and Technology Young Talents Lifting Project (20230201), and the China Apple Research System (CARS-27).

## Acknowledgments

We are grateful to Dr. Jing Zhang, Miss Wenjing Cao, and Dr. Hangkong Liu (Horticulture Science Research Center, Northwest A&F University, Yangling, China) for providing professional technical assistance. The authors declare that the research was conducted in the absence of any commercial or financial relationships that could be construed as a potential conflict of interest.

## Author contributions

M.J.L., M.R.Z., and F.W.M. conceived and supervised this study; S.J., C.H.L., C.L.Z., and L.J.D. performed the experiments; Y.C.L., L.C.Z., J.S., and X.Y.W. conducted the bioinformatics analysis; S.J. wrote the manuscript; M.J.L, B.Q.M., Y-L.R., M.R.Z., and S.J. discussed the study and revised the manuscript.

The authors responsible for the distribution of materials integral to the findings presented in this article, in accordance with the policy described in the Instructions for Authors, are: ManRang Zhang (mrz@nwsuaf.edu.cn) and Mingjun Li (limingjun@nwsuaf.edu.cn).

## Supplemental information

Document S1. Supplemental Figures 1–24

Supplemental Data 1. FPKM values of ABA-related genes in the transcriptomic data of apple roots from mycorrhizal and non-mycorrhizal plants sampled 60 days after inoculation

Supplemental Data 2. Transcriptome one: FPKM values of fatty acid-related genes in apple roots from mycorrhizal and non-mycorrhizal plants sampled 60 days after inoculation Transcriptome two: FPKM values of fatty acid-related genes in mycorrhizal roots of transgenic lines overexpressing or silencing *MdABF2*, sampled 60 days after inoculation.

Supplemental Data 3. Primer sequences used in this study

Supplemental Data 4. Gene IDs used in this study

Document S2. Article plus supplemental information

## References

[bib1] Balzergue C., Puech-Pagès V., Bécard G., Rochange S.F. (2011). The regulation of arbuscular mycorrhizal symbiosis by phosphate in pea involves early and systemic signalling events. J. Exp. Bot..

[bib2] Betz R., Heidt S., Figueira-Galán D., Hartmann M., Langner T., Requena N. (2024). Alternative splicing regulation in plants by SP7-like effectors from symbiotic arbuscular mycorrhizal fungi. Nat. Commun..

[bib3] Bennett A.E., Groten K. (2022). The costs and benefits of plant–arbuscular mycorrhizal fungal interactions. Annu. Rev. Plant Biol..

[bib4] Bernaola L., Cange G., Way M.O., Gore J., Hardke J., Stout M. (2018). Natural colonization of rice by arbuscular mycorrhizal fungi in different production areas. Rice Sci..

[bib5] Blilou I., Ocampo J.A., García-Garrido J.M. (2000). Induction of Ltp (lipid transfer protein) and Pal (phenylalanine ammonialyase) gene expression in rice roots colonized by the arbuscular mycorrhizal fungus *Glomus mosseae*. J. Exp. Bot..

[bib6] Boisson-Dernier A., Chabaud M., Garcia F., Bécard G., Rosenberg C., Barker D.G. (2001). Agrobacterium rhizogenes-transformed roots of Medicago truncatula for the study of nitrogen-fixing and endomycorrhizal symbiotic associations. Mol. Plant Microbe Interact..

[bib7] Bravo A., York T., Pumplin N., Mueller L.A., Harrison M.J. (2016). Genes conserved for arbuscular mycorrhizal symbiosis identified through phylogenomics. Nat. Plants.

[bib8] Breuillin F., Schramm J., Hajirezaei M., Ahkami A., Favre P., Druege U., Hause B., Bucher M., Kretzschmar T., Bossolini E. (2010). Phosphate systemically inhibits development of arbuscular mycorrhiza in *Petunia hybrida* and represses genes involved in mycorrhizal functioning. Plant J..

[bib9] Charpentier M., Sun J., Wen J., Mysore K.S., Oldroyd G.E.D. (2014). Abscisic acid promotion of arbuscular mycorrhizal colonization requires a component of the PROTEIN PHOSPHATASE 2A complex. Plant Physiol..

[bib10] Cutler S.R., Rodriguez P.L., Finkelstein R.R., Abrams S.R. (2010). Abscisic acid: emergence of a core signaling network. Annu. Rev. Plant Biol..

[bib11] Das D., Varshney K., Ogawa S., Torabi S., Hüttl R., Nelson D.C., Gutjahr C. (2025). Ethylene promotes SMAX1 accumulation to inhibit arbuscular mycorrhiza symbiosis. Nat. Commun..

[bib12] Depuydt S., Hardtke C.S. (2011). Hormone signalling crosstalk in plant growth regulation. Curr. Biol..

[bib13] Espinosa F., Garrido I., Ortega A., Casimiro I., Álvarez-Tinaut M.C. (2014). Redox activities and ROS, NO and phenylpropanoids production by axenically cultured intact olive seedling roots after interaction with a mycorrhizal or a pathogenic fungus. J. Plant Physiol..

[bib14] Gadkar V., David-Schwartz R., Kunik T., Kapulnik Y. (2001). Arbuscular mycorrhizal fungal colonization: factors involved in host recognition. Plant Physiol..

[bib15] Gao M., Yang N., Shao Y., Shen T., Li W., Ma B., Wei X., Ruan Y.L., Ma F., Li M. (2024). An insertion in the promoter of a malate dehydrogenase gene regulates malic acid content in apple fruit. Plant Physiol..

[bib16] Gerdemann J.W., Nicolson T.H. (1963). Spores of mycorrhizal Endogone species extracted from soil by wet sieving and decanting. Trans. Br. Mycol. Soc..

[bib17] Gutjahr C., Radovanovic D., Geoffroy J., Zhang Q., Siegler H., Chiapello M., Casieri L., An K., An G., Guiderdoni E. (2012). The half-size ABC transporters STR1 and STR2 are indispensable for mycorrhizal arbuscule formation in rice. Plant J..

[bib18] Han S., Na L., Rongchao Z., Xiuqin H., Wenyu Z., Bo Z., Xinpeng L., Zhen W., Jie X. (2023). Study on signal transmission mechanism of arbuscular mycorrhizal hyphal network against root rot of Salvia miltiorrhiza. Sci. Rep..

[bib65] He F., Zhang H., Tang M. (2016). Aquaporin gene expression and physiological responses of *Robinia pseudoacacial* L. to the mycorrhizal fungus *Rhizophagus irregularis* and drought stress. Mycorrhiza.

[bib19] Herrera-Medina M.J., Steinkellner S., Vierheilig H., Ocampo Bote J.A., García Garrido J.M. (2007). Abscisic acid determines arbuscule development and functionality in the tomato arbuscular mycorrhiza. New Phytol..

[bib20] Ivanov S., Harrison M.J. (2024). Receptor-associated kinases control the lipid provisioning program in plant-fungal symbiosis. Science.

[bib21] Jiang Y., Wang W., Xie Q., Liu N.A., Liu L., Wang D., Zhang X., Yang C., Chen X., Tang D., Wang E. (2017). Plants transfer lipids to sustain colonization by mutualistic mycorrhizal and parasitic fungi. Science.

[bib22] Jiang Y., Xie Q., Wang W., Yang J., Zhang X., Yu N., Zhou Y., Wang E. (2018). Medicago AP2-domain transcription factor WRI5a is a master regulator of lipid biosynthesis and transfer during mycorrhizal symbiosis. Mol. Plant.

[bib23] Jing S., Li Y., Zhu L., Su J., Yang T., Liu B., Ma B., Ma F., Li M., Zhang M. (2022). Transcriptomics and metabolomics reveal effect of arbuscular mycorrhizal fungi on growth and development of apple plants. Front. Plant Sci..

[bib24] Keymer A., Pimprikar P., Wewer V., Huber C., Brands M., Bucerius S.L., Delaux P.M., Klingl V., Röpenack-Lahaye E.v., Wang T.L. (2017). Lipid transfer from plants to arbuscular mycorrhiza fungi. eLife.

[bib25] Kiers E.T., Duhamel M., Beesetty Y., Mensah J.A., Franken O., Verbruggen E., Fellbaum C.R., Kowalchuk G.A., Hart M.M., Bago A. (2011). Reciprocal rewards stabilize cooperation in the mycorrhizal symbiosis. Science.

[bib26] Kobae Y., Ohmori Y., Saito C., Yano K., Ohtomo R., Fujiwara T. (2016). Phosphate treatment strongly inhibits new arbuscule development but not the maintenance of arbuscule in mycorrhizal rice roots. Plant Physiol..

[bib27] Küster H., Gutjahr C. (2017). Hormonal regulation of arbuscular mycorrhizal symbiosis: Insights from the model plant *Arabidopsis thaliana*. Plant Cell.

[bib28] Li C., Cheng X., Jia Q., Song H., Liu X., Wang K., Zhao C., Zhang Y., Ohlrogge J., Zhang M. (2017). Investigation of plant species with identified seed oil fatty acids in Chinese literature and analysis of five unsurveyed Chinese endemic species. Front. Plant Sci..

[bib29] Li X., Liu Y., He Q., Li S., Liu W., Lin C., Miao W. (2020). A candidate secreted effector protein of rubber tree powdery mildew fungus contributes to infection by regulating plant ABA biosynthesis. Front. Microbiol..

[bib30] Liao D., Wang S., Cui M., Liu J., Chen A., Xu G. (2018). Phytohormones regulate the development of arbuscular mycorrhizal symbiosis. Int. J. Mol. Sci..

[bib31] Liu C.Y., Srivastava A.K., Zhang D.J., Ying-Ning Z.O.U., Wu Q.S. (2016). Exogenous phytohormones modulate mycorrhiza-induced changes in root hair configuration of trifoliate orange. Not. Bot. Horti Agrobot. Cluj-Napoca.

[bib32] Liu J., Harrison M.J. (2020). Hormonal control of arbuscular mycorrhiza formation in plants. Plant Physiol..

[bib33] Luginbuehl L.H., Menard G.N., Kurup S., Van Erp H., Radhakrishnan G.V., Breakspear A., Oldroyd G.E.D., Eastmond P.J. (2017). Fatty acids in arbuscular mycorrhizal fungi are synthesized by the host plant. Science.

[bib34] Lumini E., Vallino M., Alguacil M.M., Romani M., Bianciotto V. (2011). Different farming and water regimes in Italian rice fields affect arbuscular mycorrhizal fungal soil communities. Ecol. Appl..

[bib35] Livak K.J., Schmittgen T.D. (2001). Analysis of relative gene expression data using real-time quantitative PCR and the 2^−ΔΔCT^ method. Methods.

[bib36] Miransari M., Abrishamchi A., Khoshbakht K., Niknam V. (2014). Plant hormones as signals in arbuscular mycorrhizal symbiosis. Crit. Rev. Biotechnol..

[bib64] Müller M., Munné-Bosch S. (2011). Rapid and sensitive hormonal profiling of complex plant samples by liquid chromatography coupled to electrospray ionization tandem mass spectrometry. Plant Methods.

[bib37] Nakashima K., Yamaguchi-Shinozaki K. (2013). ABA signaling in stress response and seed development. Plant Cell Rep..

[bib38] Peleg Z., Blumwald E. (2011). Hormone balance and abiotic stress tolerance in crop plants. Curr. Opin. Plant Biol..

[bib39] Pfeffer P.E., Douds D.D., Bécard G., Shachar-Hill Y. (1999). Carbon uptake and the metabolism and transport of lipids in an arbuscular mycorrhiza. Plant Physiol..

[bib40] Pozo M.J., López-Ráez J.A. (2020). Advances in arbuscular mycorrhizal research: The role of hormones in the regulation of the symbiosis. J. Exp. Bot..

[bib41] Rich M.K., Nouri E., Courty P.E., Reinhardt D. (2017). Diet of arbuscular mycorrhizal fungi: bread and butter?. Trends Plant Sci..

[bib42] Salzer P., Corbière H., Boller T. (1999). Hydrogen peroxide accumulation in *Medicago truncatula* roots colonized by the arbuscular mycorrhiza-forming fungus *Glomus intraradices*. Planta.

[bib43] Santner A., Estelle M. (2009). Recent advances and emerging trends in plant hormone signalling. Nature.

[bib44] Shachar-Hill Y., Pfeffer P.E., Douds D., Osman S.F., Doner L.W., Ratcliffe R.G. (1995). Partitioning of intermediate carbon metabolism in VAM colonized leek. Plant Physiol..

[bib45] Shao Y., Jiang S., Peng H., Li H., Li P., Jiang R., Fang W., Chen T., Jiang G., Yang T. (2022). Indigenous and commercial isolates of arbuscular mycorrhizal fungi display differential effects in *Pyrus betulaefolia* roots and elicit divergent transcriptomic and metabolomic responses. Front. Plant Sci..

[bib46] Shi F., Zhou X., Zhou Q., Tan Z., Yao M.M., Wei B.D., Ji S.J. (2018). Membrane lipid metabolism changes and aroma ester loss in low-temperature stored Nanguo pears. Food Chem..

[bib47] Shi J., Zhao B., Zheng S., Zhang X., Wang X., Dong W., Xie Q., Wang G., Xiao Y., Chen F. (2021). A phosphate starvation response-centered network regulates mycorrhizal symbiosis. Cell.

[bib48] Shi J., Wang X., Wang E. (2023). Mycorrhizal symbiosis in plant growth and stress adaptation: from genes to ecosystems. Annu. Rev. Plant Biol..

[bib49] Smith S.E., Smith F.A. (2011). Roles of arbuscular mycorrhizas in plant nutrition and growth: new paradigms from cellular to ecosystem scales. Annu. Rev. Plant Biol..

[bib50] Shin H., Ohlrogge J.B. (2009). Acetyl-CoA carboxylase is a key regulatory enzyme in plant lipid metabolism. Curr. Opin. Plant Biol..

[bib51] Trépanier M., Bécard G., Moutoglis P., Willemot C., Gagné S., Avis T.J., Rioux J.A. (2005). Dependence of arbuscular-mycorrhizal fungi on their plant host for palmitic acid synthesis. Appl. Environ. Microbiol..

[bib52] Vallino M., Greppi D., Novero M., Bonfante P., Lupotto E. (2009). Rice root colonisation by mycorrhizal and endophytic fungi in aerobic soil. Ann. Appl. Biol..

[bib53] Vanstraelen M., Benková E. (2012). Hormonal interactions in the regulation of plant development. Annu. Rev. Cell Dev. Biol..

[bib54] Wang S., Han L., Ren Y., Hu W., Xie X., Chen H., Tang M. (2024). The receptor kinase RiSho1 in *Rhizophagus irregularis* regulates arbuscule development and drought tolerance during arbuscular mycorrhizal symbiosis. New Phytol..

[bib55] Yang S., Zhou J., Li Y., Wu J., Ma C., Chen Y., Sun X., Wu L., Liang X., Fu Q. (2023). AP2/EREBP pathway plays an important role in chaling wild rice tolerance to cold stress. Int. J. Mol. Sci..

[bib56] Yeap W.C., Lee F.C., Shabari Shan D.K., Musa H., Appleton D.R., Kulaveerasingam H. (2017). WRI 1-1, ABI 5, NF-YA3, and NF-YC2 increase oil biosynthesis in coordination with hormonal signaling during fruit development in oil palm. Plant J..

[bib57] Zhang M., Smith J.A.C., Harberd N.P., Jiang C. (2016). The regulatory roles of ethylene and reactive oxygen species (ROS) in plant salt stress responses. Plant Mol. Biol..

[bib58] Zhang Q., Blaylock L.A., Harrison M.J. (2010). Two *Medicago truncatula* half-ABC transporters are essential for arbuscule development in arbuscular mycorrhizal symbiosis. Plant Cell.

[bib59] Zhang Q., Wang S., Xie Q., Xia Y., Lu L., Wang M., Wang G., Long S., Cai Y., Xu L. (2023). Control of arbuscule development by a transcriptional negative feedback loop in symbiosis. Nat. Commun..

[bib60] Zheng X., Zhao Y., Shan D., Shi K., Wang L., Li Q., Wang N., Zhou J., Yao J., Xue Y. (2018). MdWRKY9 overexpression confers intensive dwarfing in the M26 rootstock of apple by directly inhibiting brassinosteroid synthetase MdDWF4 expression. New Phytol..

[bib61] Zhou Q., Ravnskov S., Jiang D., Wollenweber B. (2015). Changes in carbon and nitrogen allocation, growth and grain yield induced by arbuscular mycorrhizal fungi in wheat (*Triticum aestivum* L.) subjected to a period of water deficit. Plant Growth Regul..

[bib62] Zhu L., Li Y., Wang C., Wang Z., Cao W., Su J., Peng Y., Li B., Ma B., Ma F. (2023). The SnRK2.3-AREB1-TST1/2 cascade activated by cytosolic glucose regulates sugar accumulation across tonoplasts in apple and tomato. Nat. Plants.

