# Supplementary material for: ABA promotes fatty acid biosynthesis and transport to boost arbuscular mycorrhizal symbiosis in apple roots
Source: Plant Commun. 2025 Jun 24;6(8):101426. doi: 10.1016/j.xplc.2025.101426 (PMC12365809; doi:10.1016/j.xplc.2025.101426)
Supplement: Document S1. Supplemental Figures 1–24 [file mmc1.pdf]

Plant Communications, Volume *6*

Supplemental information

ABA promotes fatty acid biosynthesis and transport to boost arbuscular mycorrhizal symbiosis in apple roots

Shan Jing, Mingjun Li, Chunhui Li, Chunlei Zhang, Lingcheng Zhu, Lijun Du, Yuchao Li, Xiaoyu Wei, Manrang Zhang, Baiquan Ma, Yongling Ruan, and Fengwang Ma

---

ABA promotes fatty acid biosynthesis and transport to boost arbuscular mycorrhizal symbiosis in apple roots

Shan Jing, Mingjun Li*, Chunhui Li, Chunlei, Zhang, Lingcheng Zhu，Lijun Du, Yuchao Li, Xiaoyu Wei, Manrang Zhang*, Baiquan Ma, Yongling Ruan, Fengwang Ma

State Key Laboratory for Crop Stress Resistance and High-Efficiency Production / Shaanxi Key Laboratory of Apple, College of Horticulture, Northwest A&F University, Yangling 712100, Shaanxi, China.

***Corresponding authors:**

Mingjun Li

E-mail address: limingjun@nwsuaf.edu.cn

Tel: 86-029-87082613

Manrang Zhang

E-mail address: mrz@nwsuaf.edu.cn

Tel: 86-029-87082613

This supplementary file includes the parts of 24 supplementary figures and 4 supplementary data sets.

Supplementary Data 1. FPKM values of ABA-related genes in the transcriptomic data of the apple roots of mycorrhizal and non-mycorrhizal plants that were sampled after inoculation for 60 days.

Supplementary Data 2. transcriptome one FPKM values of fatty acid-related genes in the transcriptomic data of the apple roots of mycorrhizal and non-mycorrhizal plants that were sampled after inoculation for 60 days.

Supplementary Data 2. transcriptome two FPKM values of fatty acid-related genes in the transcriptomic data of mycorrhizal roots of transgenic lines overexpressing or silencing MdABF2 that were sampled after inoculation for 60 days.

Supplementary Data 3. Primer sequences used in this study.

Supplementary Data 4. All gene IDs used in this study.

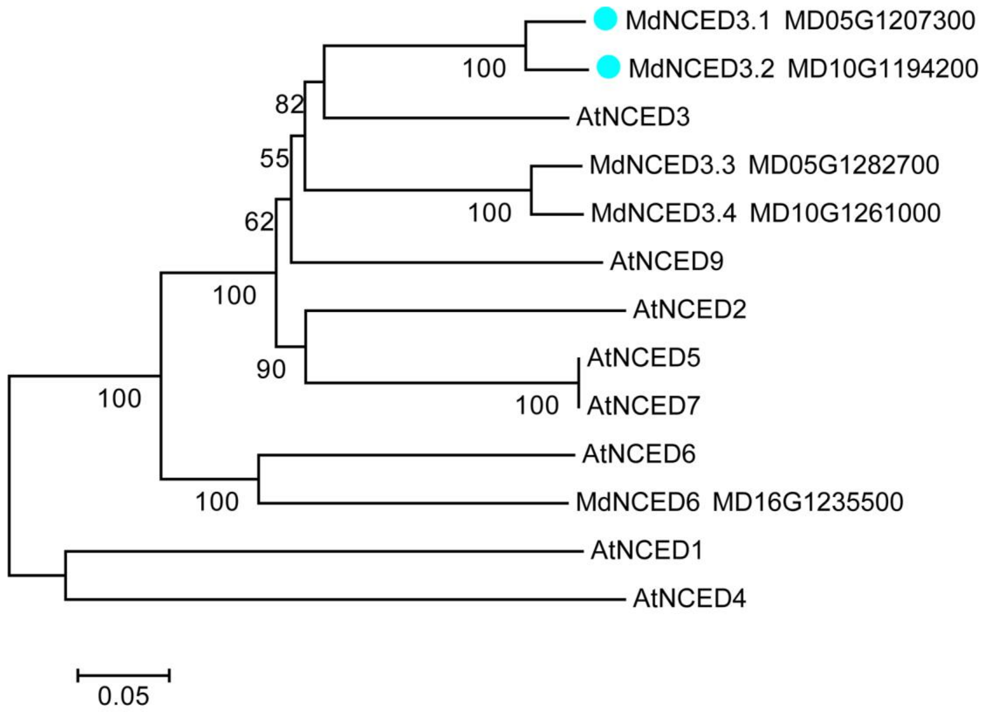

Supplemental Figure 1. Phylogenetic analysis of 9-cis-epoxycarotenoid dioxygenase (NCED) proteins from apple (*Malus* × *domestica*) and *Arabidopsis thaliana*. The phylogenetic tree was constructed using the maximum likelihood method of the MEGA7 software. Bootstrap analysis of 1000 trials provided a reliable estimate of the topology of the phylogenetic tree. The blue dots represent candidate genes for further research.

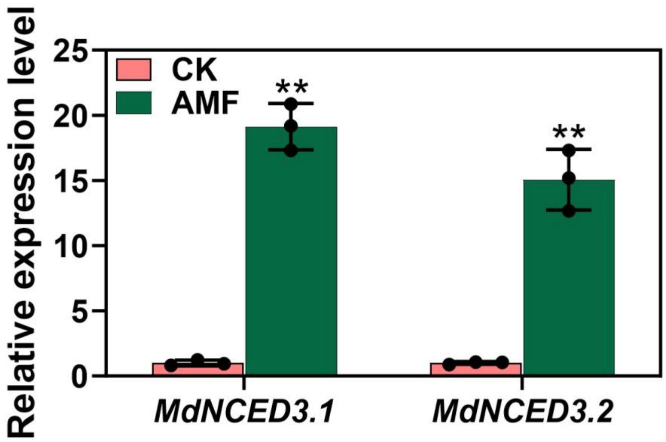

Supplemental Figure 2. The relative expressi on levels of *MdNCED3.1* and *MdNCED3.2* in the roots of 60-day-old uninoculated M26 (*Malus pumila* Mill.) plants and M26 plants inoculated with AMF. The transcript levels were normalized to those of *MdActin*. Relative expression levels for each gene were obtained via the ddCT method, with its expression in uninoculated M26 plants set as ‘1’. The bars represent the mean value ± SD (n = 3, representing independent biological replicates). Mixed samples from three M26 plants were as one replicate. The asterisks indicate significant differences as assessed by one-way ANOVA (two-sided Student's t-test; ** $P < 0.01$).

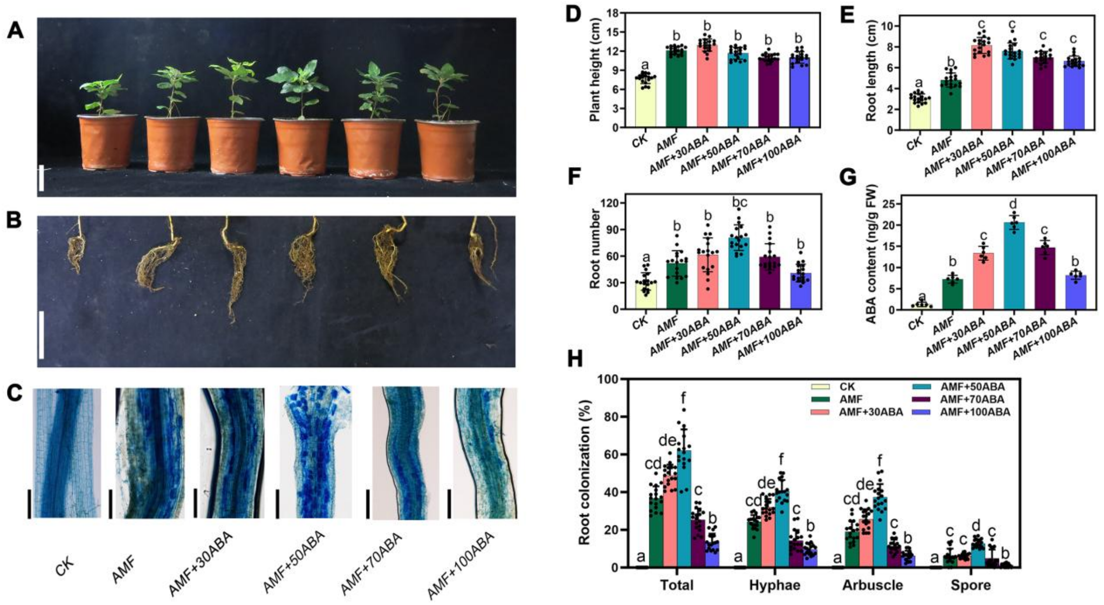

Supplemental Figure 3. The effects of exogenous ABA at different concentrations on AM symbiosis in M26 apple (*Malus pumila* Mill.) plants.

(**A–C**) Phenotypes of 45-day-old M26 plants under control (CK), arbuscular mycorrhizae-inoculated (AMF), and arbuscular mycorrhizae-inoculated plus different concentration ABA-treated (AMF+ABA) conditions. (A) Plant height. Scale bars, 5 cm. (**B**) Root structure. Scale bars, 5 cm. (**C**) Trypan blue staining of the fungus to reveal arbuscule morphology. Scale bars, 100 μm. (**D**) Plant height of M26 plants. (**E**) Root length of M26 plants. (**F**) Root number of M26 plants. (**G**) ABA content in the roots of M26 plants. The bars represent the mean value ± SD (*n* = 6, representing independent biological replicates). Mixed samples from three M26 plants were as one replicate. (**H**) Quantification of mycorrhizal colonization levels. FW, fresh weight; CK, Uninoculated plants without ABA treatment for 45 days, as control. AMF, *Rhizophagus irregularis*-inoculated plants without ABA treatment for 45 days. AMF+30ABA, AMF+50ABA, AMF+70ABA, and AMF+100ABA, *Rhizophagus irregularis*-inoculated plants were sprayed respectively with exogenous ABA (30 μmol/L, 50 μmol/L, 70 μmol/L and 100 μmol/L) for 15 days after inoculation for 30 days. (D, E, F, and H) The bars represent the mean value ± SD (*n* = 18, representing independent biological replicates). (**D**, **E**, **F, G**, and **H)** Different letters indicate significant difference (analysis of variance [ANOVA]), Duncan's multiple range test; P < 0.05).

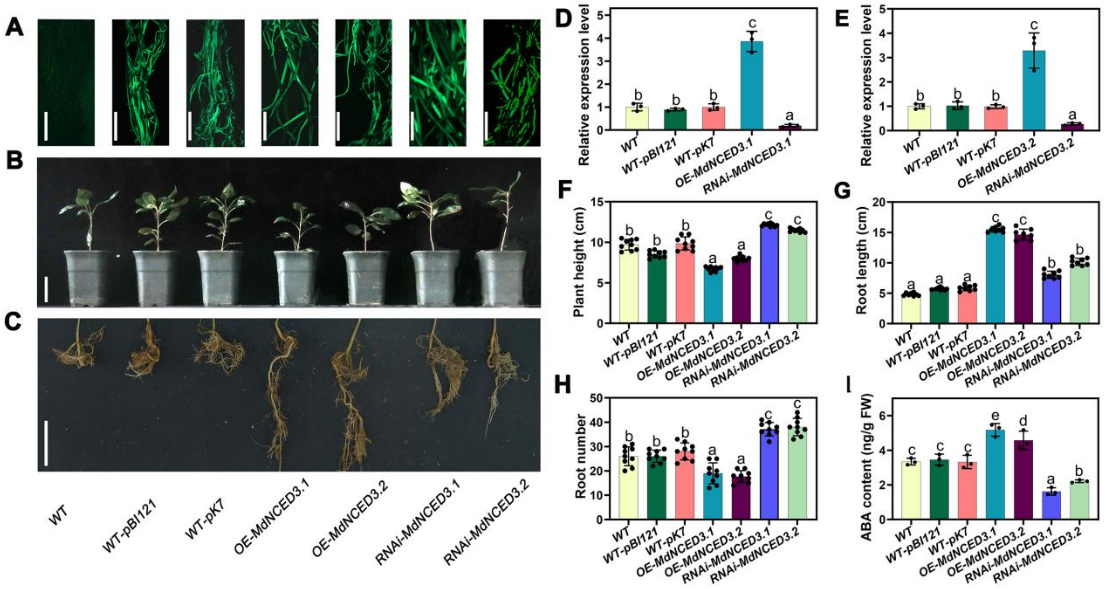

Supplemental Figure 4. The effects of changing the expression of *MdNCED3.1*/*3.2* in the roots of 60-day-old apple (*Malus hupehensis* Rhed) seedlings without inoculated AMF. Each construct contained a green fluorescent protein (GFP) cassette for screening the transformation. **(A)** Images of the transgenic root systems of apple seedlings with GFP. Scale bars, 1 mm. **(B)** Above-ground growth phenotypes of transgenic hairy root apple lines. Scale bars, 5 cm. **(C)** Root growth phenotypes of transgenic hairy root apple lines. Scale bars, 5 cm. **(D)** Relative expression levels of *MdNCED3.1* mRNA in transgenic apple roots to the WT control (set as ‘1’). **(E)** Relative expression levels of *MdNCED3.2* mRNA in transgenic apple roots compared to the WT control (set as ‘1’). **(F)** Plant height of apple seedlings carrying transgenic hairy roots. **(G)** Root length of apple seedlings carrying transgenic hairy roots. **(H)** Root number of apple seedlings carrying transgenic hairy roots. **(I)** The ABA content in the root of apple transgenic lines. FW, fresh weight; WT, wild type; WT-pBI121, Apple seedlings transformed with an hairy root empty vector for overexpression and containing the GFP tag (plasmid Binary Vector 121); WT-pK7, Apple seedlings transformed with an RNA interference empty vector containing the GFP tag (pK7GWIWG2); OE-*MdNCED3.1*, *MdNCED3.1*-overexpressing root lines; OE-*MdNCED3.2*, *MdNCED3.2*-overexpressing root lines; RNAi-*MdNCED3.1*, *MdNCED3.1*-RNA interference root lines; RNAi-*MdNCED3.2*, *MdNCED3.2*-RNA interference root lines. **(D**, **E**, and **I)** The bars represent the mean value ± SD (n = 3, representing independent biological replicates). Samples from three carrying transgenic hairy roots were as one replicate. (**F**, **G,** and **H)** The bars represent the mean value ± SD (n = 9, representing independent biological replicates). (**D, E**, **F**, **G, H,** and **I)** Different letters indicate significant difference (analysis of variance [ANOVA]), Duncan's multiple range test; $P < 0.05$).

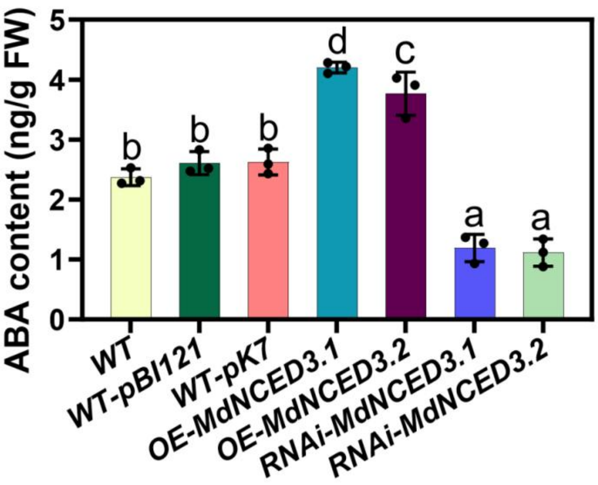

Supplemental Figure 5. The ABA content was non-treated with *MdNCED3.1/3.2* transgenic hairy roots in apple root. FW, fresh weight; WT, wild type; WT-pBI121, Apple seedlings transformed with an overexpressed empty vector containing the GFP tag (plasmid Binary Vector 121); WT-pK7, Apple seedlings transformed with RNA interference empty vector containing the GFP tag (pK7GWIWG2); OE-*MdNCED3.1*, *MdNCED3.1*-overexpressing root lines; OE-*MdNCED3.2*, MdNCED3.2-overexpressing root lines; RNAi-*MdNCED3.1*, *MdNCED3.1-RNA* interference root lines; RNAi-*MdNCED3.2*, *MdNCED3.2*-RNA interference root lines. The bars represent the mean value ± SD (n = 3, representing independent biological replicates). Mixed samples from three apple seedlings carrying transgenic hairy roots were as one replicate. Different letters indicate significant difference (analysis of variance [ANOVA]), Duncan's multiple range test; $P < 0.05$).

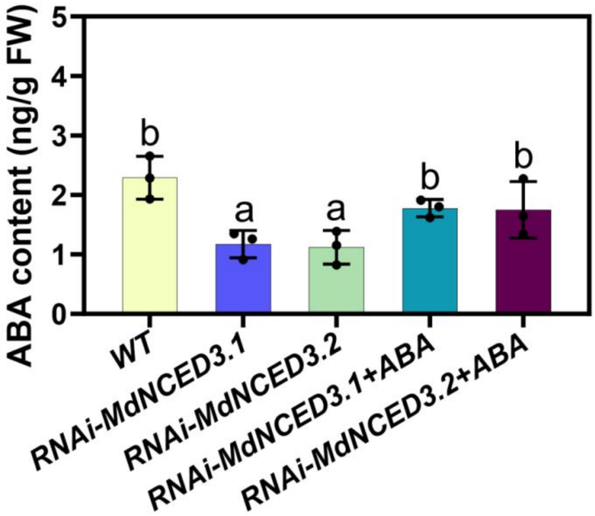

Supplemental Figure 6. The ABA content with *MdNCED* transgenic hairy roots in apple root. FW, fresh weight; WT, wild type; RNAi-*MdNCED3.1*, *MdNCED3.1*-RNA interference root lines; RNAi-*MdNCED3.1* +ABA indicates *MdNCED3.1*-RNA interference roots that were sprayed with exogenous ABA (50 µmol/L) for 15 days; RNAi-*MdNCED3.2*, *MdNCED3.2*-RNA interference root lines; RNAi-*MdNCED3.2*+ABA indicates *MdNCED3.2*-RNA interference roots that were sprayed with exogenous ABA (50 µmol/L) for 15 days. The bars represent the mean value ± SD (n = 3, representing independent biological replicates). Mixed samples from three apple seedlings carrying transgenic hairy roots were as one replicate. Different letters indicate significant difference (analysis of variance [ANOVA]), Duncan's multiple range test; $P < 0.05$).

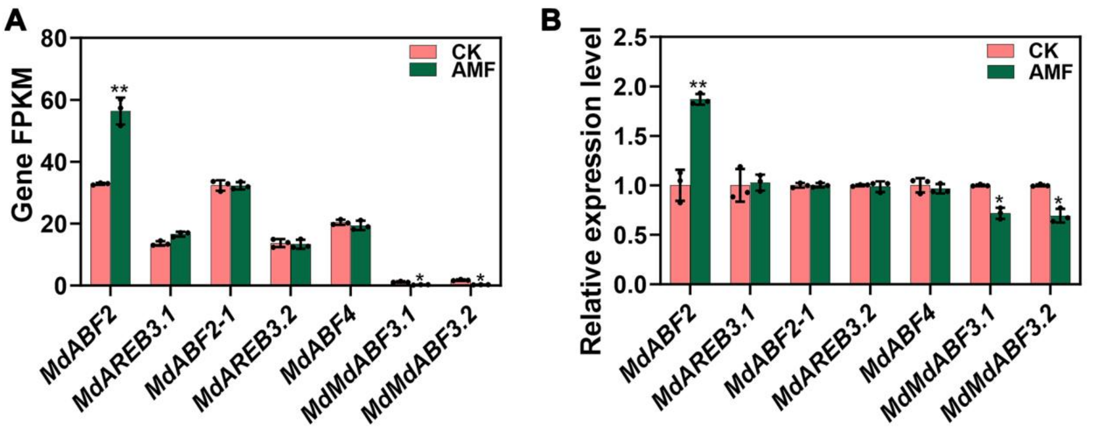

Supplemental Figure 7. (A) RNAseq-based FPKM levels of *MdABF*/*MdAREB* expression in the roots of 60-day-old inoculated M26 (*Malus pumila* Mill.) plants were compared to those in uninoculated M26 plants. **(B)** The qPCR-based relative expression levels of *MdABF*/*MdAREB* in the roots of 60-day-old inoculated M26 (*Malus pumila* Mill.) plants were compared to those in uninoculated M26 plants. The transcript levels were normalized to those of *MdActin*. Relative expression levels for each gene were obtained via the ddCT method, with its expression in uninoculated M26 plants set as '1'. The bars represent the mean value ± SD (n = 3, representing independent biological replicates). Mixed samples from three M26 plants were as one replicate. The asterisks indicate significant differences as assessed by one-way ANOVA (two-sided Student's t-test; **$P < 0.01$, *$P < 0.05$).

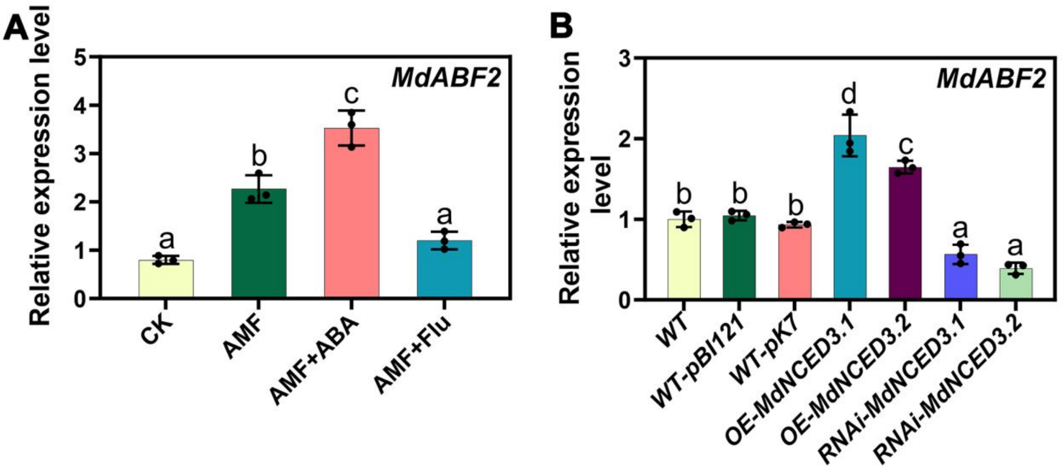

Supplemental Figure 8. Relative expression levels of *MdABF2* in apple roots following *R. irregularis* infection. (A) Relative expression levels of *MdABF2* in the roots of 45-day-old M26 (*Malus pumila* Mill.) plants under CK, AMF, AMF+ABA, and AMF+Flu conditions, as determined by qPCR, compared to the CK (set as '1'). CK, Un-inoculated plants without ABA treatment for 45 days as control. AMF, Mycorrhizal-inoculated plants without ABA treatment for 45 days. AMF+ABA, Mycorrhizal-inoculated plants were grown for 30 days before spraying with exogenous ABA (50 µmol/L) and further growth for 15 days. AMF+ Flu, mycorrhizal-inoculated plants were grown for 30 days before spraying with exogenous Flu (50 µmol/L ABA synthesis inhibitor fluoridone) and further growth for 15 days. The bars represent the mean value ± SD (*n* = 3, representing independent biological replicates). Samples from three M26 plants were as 1 replicate. **(B)** The relative expression levels, based on qPCR, of MdABF2 (*Malus hupehensis* Rhed) in the roots of 60-day-old WT-pBI121, WT-pK7, *O*E-*MdNCED3.1*, OE-*MdNCED3.2*, RNAi-*MdNCED3.1*, and RNAi-*MdNCED3.2* apple seedlings with transgenic hairy roots, compared to untransformed WT controls (set as '1'). The bars represent the mean value ± SD (n = 3, representing independent biological replicates). Mixed samples from three apple seedlings carrying transgenic hairy roots were as one replicate. The transcript levels were normalized to those of *MdActin*. Relative expression levels for each gene were obtained via the ddCT method. Different letters indicate significant difference (analysis of variance [ANOVA]), Duncan's multiple range test; $P < 0.05$).

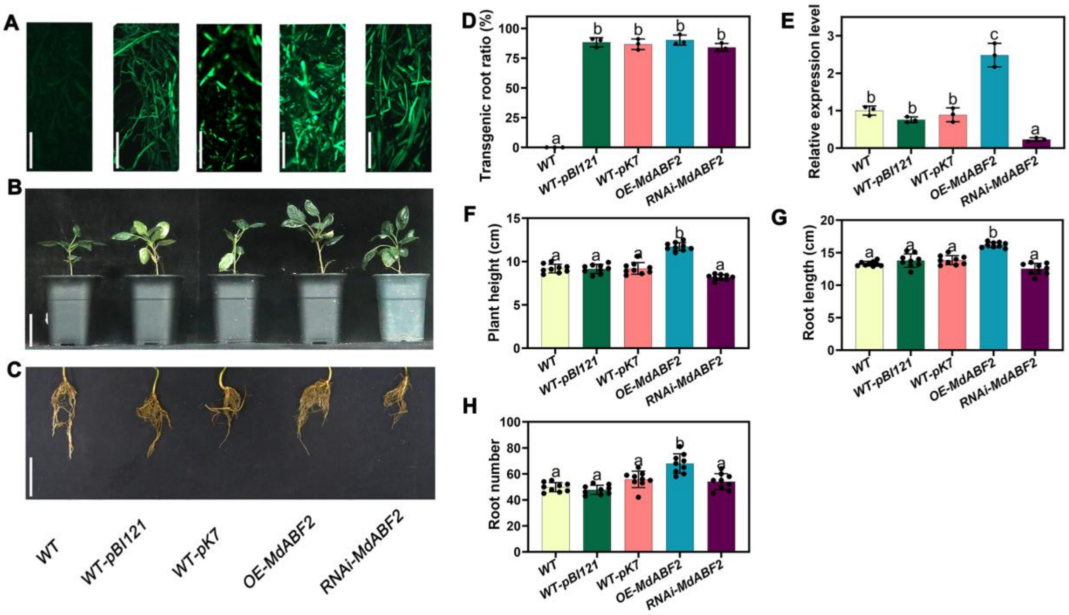

Supplemental Figure 9. The effects of changing the expression of *MdABF2* in the roots of 60-day-old apple (*Malus hupehensis* Rhed) seedlings not inoculated with AMF. Each construct contained a GFP cassette for screening the transformation. (A) Images of the transgenic root systems of apple seedlings expressing green fluorescent protein. Scale bars, 1 mm. **(B)** Above-ground growth phenotypes of apple seedlings carrying transgenic hairy roots. Scale bars, 5 cm. **(C)** Root growth phenotypes of apple seedlings carrying transgenic hairy roots. Scale bars, 5 cm. **(D)** Transgenic root ratio of apple seedlings carrying transgenic hairy roots after root co-culture with *Agrobacterium rhizogenes*. **(E)** The relative expression levels of *MdABF2* mRNA in the root of the apple seedlings carrying transgenic hairy roots with the indicated constructs compared to the WT control (set as '1'). The bars represent the mean value ± SD (n = 3, representing independent biological replicates). Mixed samples from three apple seedlings carrying transgenic hairy roots were as one replicate. **(F)** Plant height of apple seedlings carrying transgenic hairy roots. **(G)** Root length of apple seedlings carrying transgenic hairy roots. **(H)** Root number of apple seedlings carrying transgenic hairy roots. WT, wild type; WT-pBI121, apple seedlings transformed with an empty overexpression vector containing the GFP tag (plasmid Binary Vector 121); WT-pK7, apple seedlings transformed with an empty RNA interference vector containing the GFP tag (pK7GWIWG2); OE-*MdABF2*, *MdABF2*-overexpressing root lines; RNAi-*MdABF2*, *MdABF2*-RNA interference root lines. (**D**, **F**, **G**, and **H)** The bars represent the mean value ± SD (n = 9, representing independent biological replicates). ((**D**, **E**, **F**, **G**, and **H)** Different letters indicate significant difference (analysis of variance [ANOVA]), Duncan's multiple range test; $P < 0.05$).

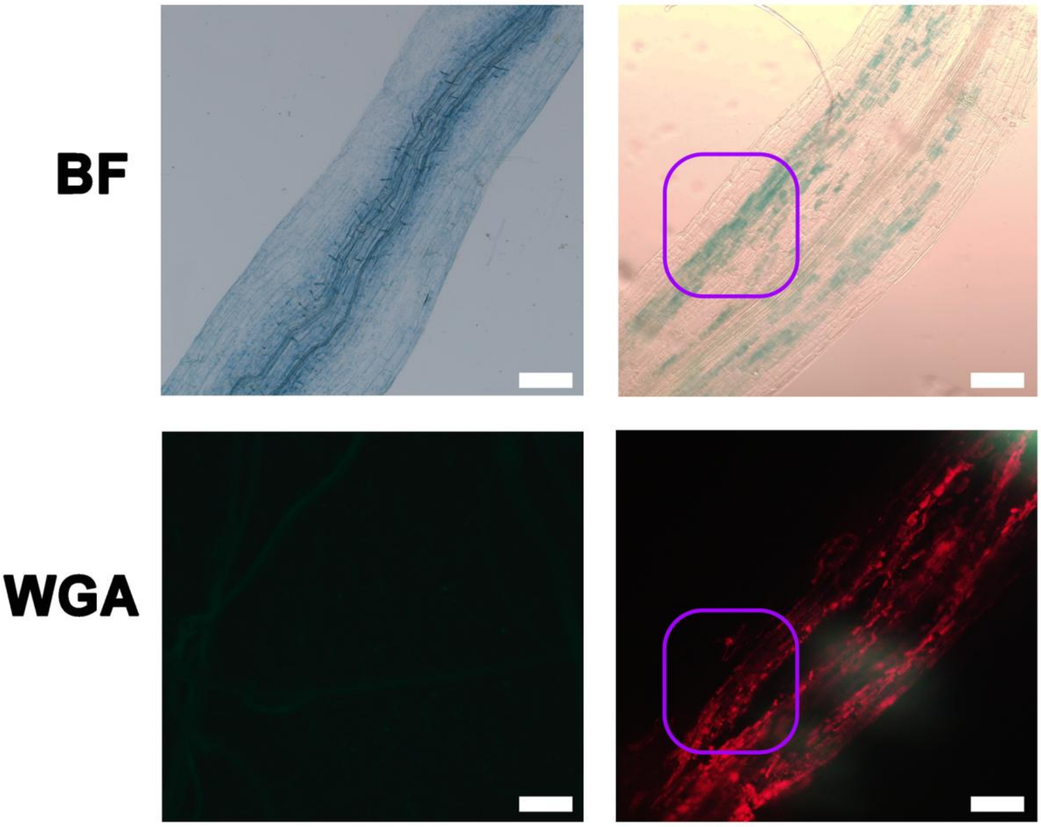

Supplemental Figure 10. GUS expression is driven by the MdABF2 promoter in the apple root system. Scale bars, 100 μm. The microscopy images show bright field images (up) and corresponding fluorescence images (down). The microscopy images of roots show both the inoculation with *R. regularis* (right) and the uninoculation with *R. regularis* (left). WGA, WGA-Alexa Fluor 633; BF, Bright Field. The part circled in pink represents the overlapping portion of the bright field and the fluorescence field.

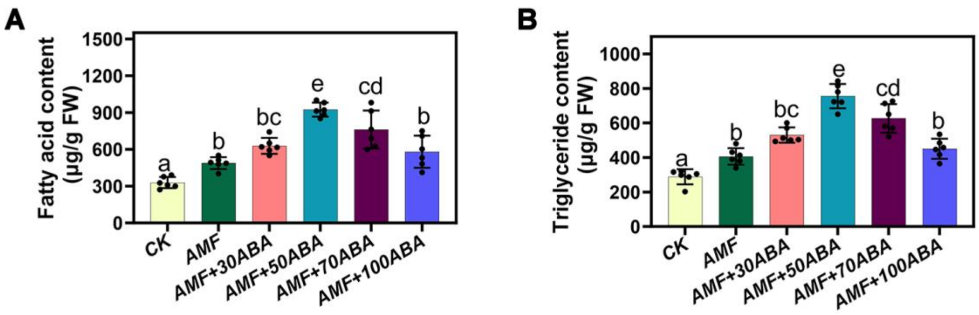

Supplemental Figure 11. Fatty acid and triglyceride contents in apple roots following *R. irregularis* infection. (A and B) M26 apple rootstock roots were tested for fatty acid **(A)** and triglyceride **(B)** content under CK, AMF, AMF+30ABA, AMF+50ABA, AMF+70ABA, and AMF+100ABA conditions. FW, fresh weight; CK, Uninoculated plants without ABA treatment for 45 days, as control. AMF, *Rhizophagus irregularis*-inoculated plants without ABA treatment for 45 days. AMF+30ABA, AMF+50ABA, AMF+70ABA, and AMF+100ABA, *Rhizophagus irregularis*-inoculated plants were sprayed respectively with exogenous ABA (30 μmol/L, 50 μmol/L, 70 μmol/L and 100 μmol/L) for 15 days after inoculation for 30 days. (A and B) The bars represent the mean value ± SD (n = 6, representing independent biological replicates). Mixed samples from three M26 plants were as one replicate. Different letters indicate significant difference (analysis of variance [ANOVA]), Duncan's multiple range test; $P < 0.05$).

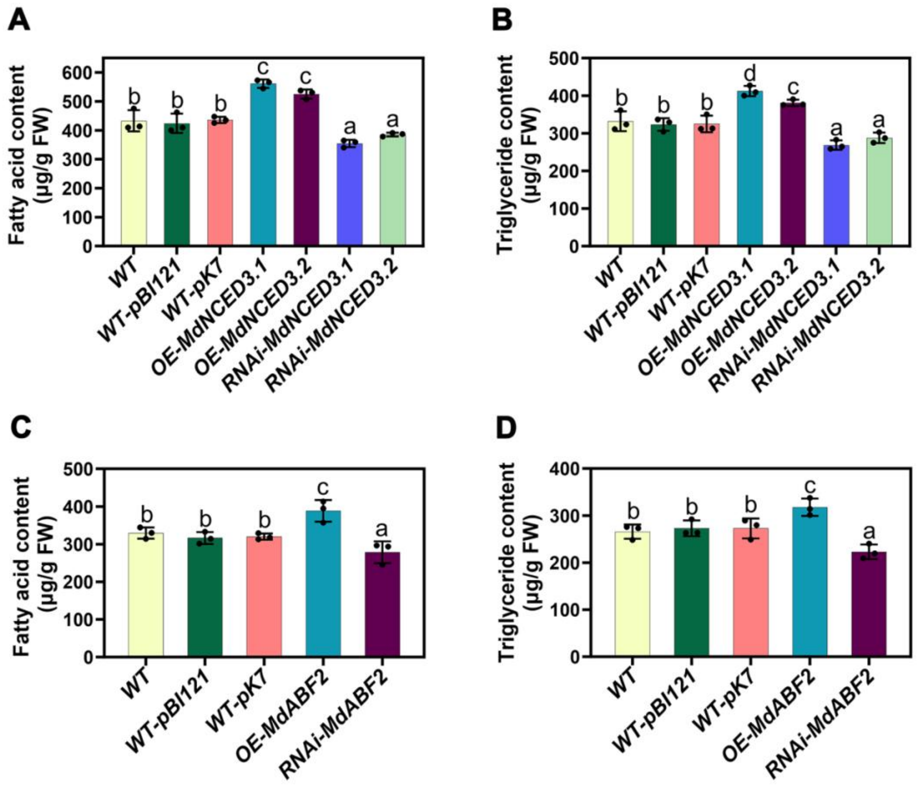

Supplemental Figure 12. Fatty acid and triglyceride content in the roots of 60-day-old apple (*Malus hupehensis* Rhed) seedlings carrying transgenic hairy roots without *R. irregularis* infection of the roots. (A), (B) Fatty acid (**A**) and triglyceride (**B**) content of WT, WT-pBI121, WT-pK7, OE-*MdNCED3.1*, OE-*MdNCED3.2*, RNAi-*MdNCED3.1*, and RNAi-*MdNCED3.2* apple seedlings with transgenic hairy roots, compared to untransformed WT controls. **(C), (D)** Fatty acid (**C**) and triglyceride (**D**) content of WT, WT-pBI121, WT-pK7, OE-*MdABF2*, and RNAi-*MdABF2* apple transgenic hairy roots. FW, fresh weight. The bars represent the mean value ± SD (n = 3, representing independent biological replicates). Mixed samples from three apple seedlings carrying transgenic hairy roots were as one replicate. Different letters indicate significant difference (analysis of variance [ANOVA]), Duncan's multiple range test; $P < 0.05$).

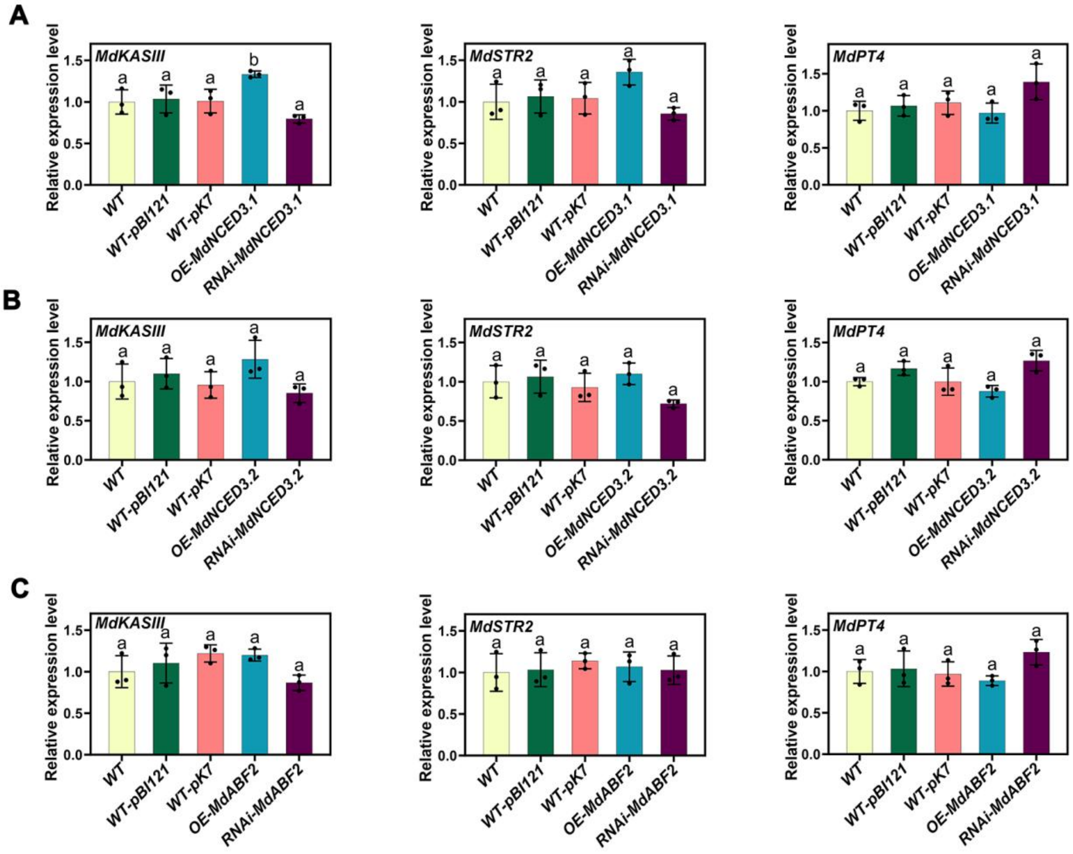

Supplemental Figure 13. The relative gene expression levels in the roots of 60-day-old apple (*Malus hupehensis* Rhed) transgenic seedlings without *R. irregularis* infection of the transgenic roots. (A) The relative expression level of *MdKASIII***,** *MdSTR2* and *MdPT4* based on qPCR in WT, WT-pBI121, WT-pK7, OE-*MdNCED3.1*, and RNAi-*MdNCED3.1* apple transgenic hairy roots. **(B)** The relative expression level of *MdKASIII***,** *MdSTR2* and *MdPT4* based on qPCR in WT, WT-pBI121, WT-pK7, OE-*MdNCED3.2*, and RNAi-*MdNCED3.2* apple transgenic hairy roots. **(C)** The relative expression level of *MdKASIII***,** *MdSTR2* and *MdPT4* based on qPCR in WT, WT-pBI121, WT-pK7, OE-*MdABF2*, and RNAi-*MdABF2* apple transgenic hairy roots. Relative expression levels for each gene were obtained via the ddCT method, with its expression in WT set as ‘1’. The bars represent the mean value ± SD (n = 3, representing independent biological replicates). Mixed samples from three apple seedlings carrying transgenic hairy roots were as one replicate. Different letters indicate significant difference (analysis of variance [ANOVA]), Duncan's multiple range test; $P < 0.05$).

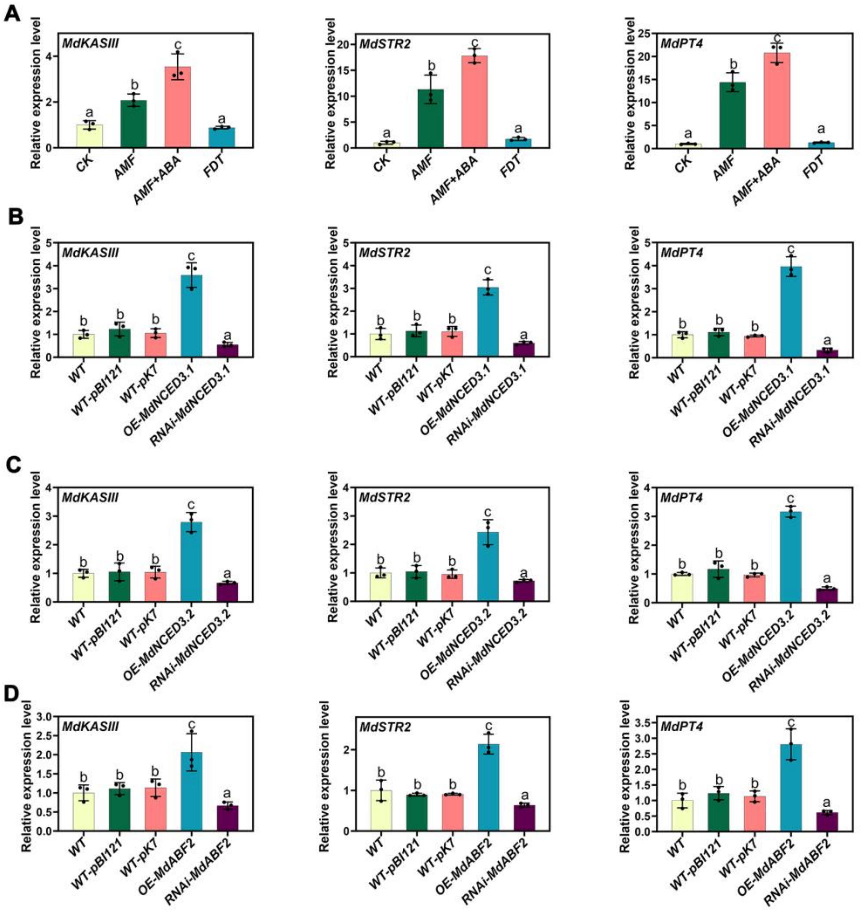

Supplemental Figure 14. The relative gene expression levels in apple roots following *R. irregularis* infection. (A) The relative expression level of *MdKASIII*, *MdSTR2* and *MdPT4* based on qPCR in non-transgenic M26 apple rootstock roots under CK, AMF, AMF+ABA, and AMF+Flu conditions. CK, Non-mycorrhizal inoculated plants without ABA treatment for 45 days as a control. AMF, Mycorrhizal-inoculated plants without ABA treatment for 45 days. AMF+ABA, Mycorrhizal-inoculated plants were prayed with exogenous ABA (50 μmol/L) for 15 days after inoculation for 30 days. AMF+ Flu, mycorrhizal-inoculated plants were prayed with exogenous Flu (50 μmol/L ABA synthesis inhibitor fluoridone) for 15 days after inoculation for 30 days. **(B)** The relative expression level of *MdKASIII*, *MdSTR2* and *MdPT4* based on qPCR in WT, WT-pBI121, WT-pK7, OE-*MdNCED3.1*, and RNAi-*MdNCED3.1* apple transgenic hairy roots.
(C) The relative expression level of *MdKASIII*, *MdSTR2* and *MdPT4* based on qPCR in WT, WT-pBI121, WT-pK7, OE-*MdNCED3.2*, and RNAi-*MdNCED3.2* apple transgenic hairy roots.
(D) The relative expression level of *MdKASIII*, *MdSTR2* and *MdPT4* based on qPCR in WT, WT-pBI121, WT-pK7, OE-*MdABF2*, and RNAi-*MdABF2* apple transgenic hairy roots. (A) Relative expression levels for each gene were obtained via the ddCT method, with its expression in CK set as '1'. (B, C, D) Relative expression levels for each gene were obtained via the ddCT method, with its expression in WT set as '1' (*n* = 3, representing independent biological replicates). (A) Mixed samples from three M26 plants were as one replicate. (B, C, D) Mixed samples from three apple seedlings carrying transgenic hairy roots were as one replicate. Different letters indicate significant difference (analysis of variance [ANOVA]), Duncan's multiple range test; $P < 0.05$).

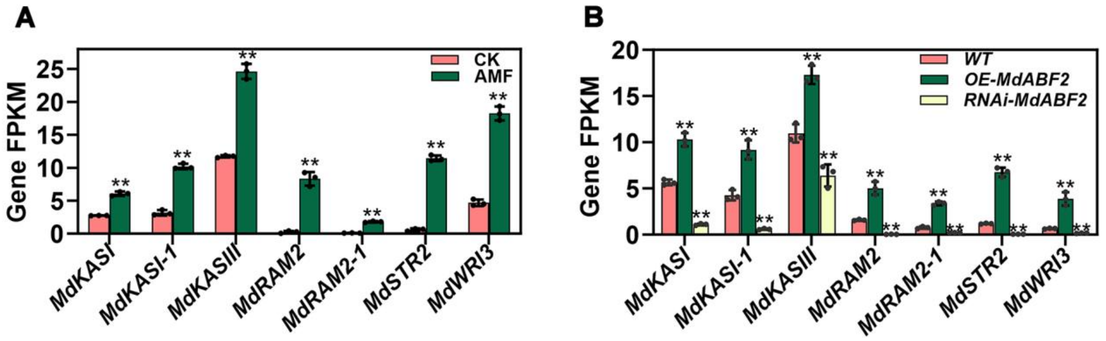

Supplemental Figure 15. FPKM values for common fatty acid-associated genes that were differentially expressed at a significant level between two transcriptomes. (A) The FPKM values of differential gene expression levels related to fatty acids based on RNA-seq in the roots of 60-day-old uninoculated and inoculated M26 (*Malus pumila* Mill.) plants. **(B)** The FPKM values of differential gene expression levels related to fatty acids based on RNA-seq in WT and the OE-*MdABF2* apple (*Malus hupehensis* Rhed) transgenic hairy roots or the RNAi-*MdABF2* apple transgenic hairy roots. Expression-fold changes of DEGs (|log 2 FC| > 1, FDR < 0.05) represented significant difference. The bars represent the mean value ± SD (n = 3, representing independent biological replicates). Mixed samples from three apple seedlings carrying transgenic hairy roots were as one replicate. The asterisks indicate significant differences as assessed by one-way ANOVA (two-sided Student's t-test; **P < 0.01).

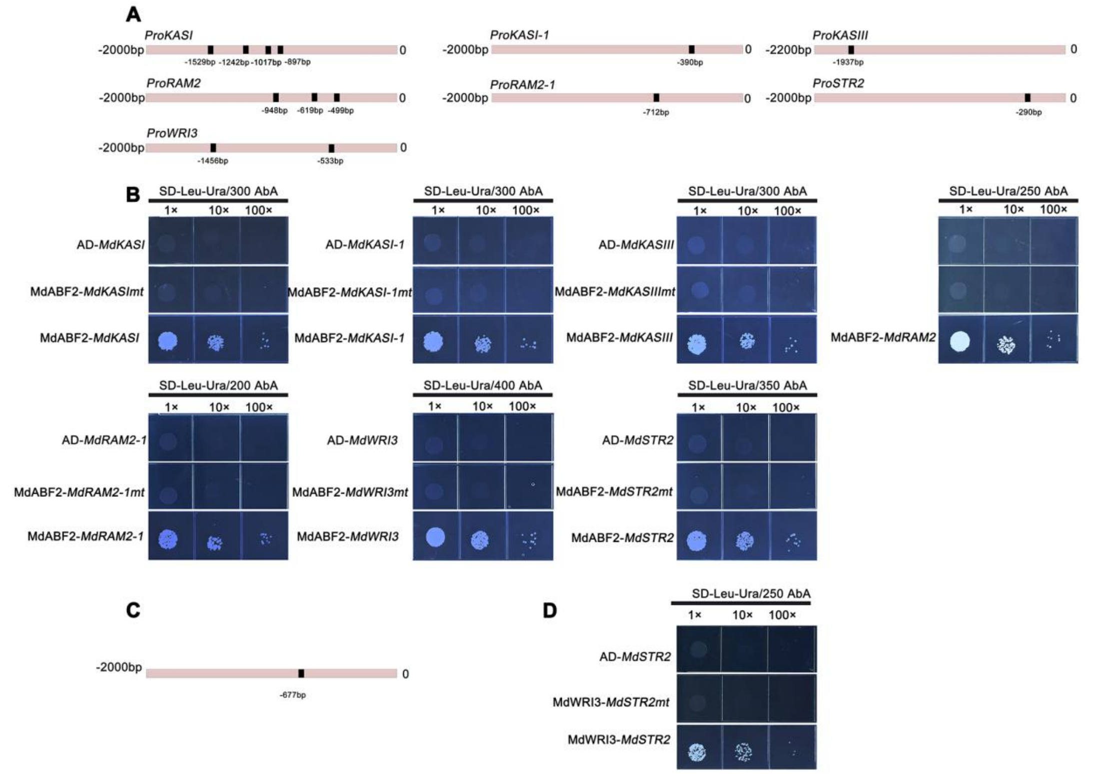

Supplemental Figure 16. ABF2 interacts with the *MdKASI*, *MdKASI-1*, *MdKASIII*, *MdRAM2*, *MdRAM2-1*, *MdSTR2*, and *MdWRI3* promoters in a yeast one-hybrid assay. WRI3 interacts with the *MdSTR2* promoter in a yeast one-hybrid assay. (A) Locations of predicted ABRE in the *MdKASI*, *MdKASI-1*, *MdKASIII*, *MdRAM2*, *MdRAM2-1*, *MdSTR2*, and *MdWRI3* promoters. **(B)** The Y1H assays demonstrated that MdABF2 directly binds to the *MdKASI*, *MdKASI-1*, *MdKASIII*, *MdRAM2*, *MdRAM2-1*, *MdSTR2*, and *MdWRI3* promoters. **(C)** Locations of predicted AW-box in the *MdSTR2* promoter. **(D)** The Y1H assays demonstrated that MdWRI3 directly binds to the *MdSTR2* promoter. *pGADT7* (AD) was used as a negative control. mt represents mutating all transcription factor binding sites in the promoter region of the target gene.

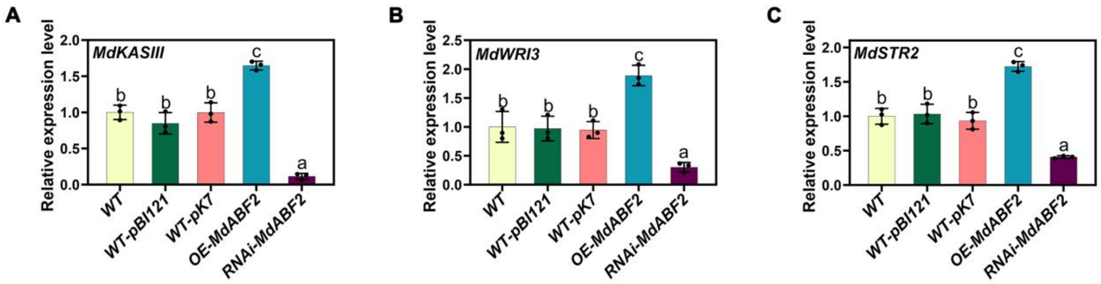

Supplemental Figure 17. The relative gene expression levels in the roots of 60-day-old apple (*Malus hupehensis* Rhed) transgenic seedlings with *R. irregularis* infection of the transgenic roots. (A–C) The relative expression level of *MdKASIII* **(A),** *MdWRI3* **(B),** and *MdSTR2* **(C)** based on qPCR in WT, WT-pBI121, WT-pK7, OE-*MdABF2*, and RNAi-*MdABF2* apple transgenic hairy roots. Relative expression levels for each gene were obtained via the ddCT method, with its expression in WT set as '1'. The bars represent the mean value ± SD (n = 3, representing independent biological replicates). Mixed samples from three apple seedlings carrying transgenic hairy roots were as one replicate. Different letters indicate significant difference (analysis of variance [ANOVA]), Duncan's multiple range test; $P < 0.05$).

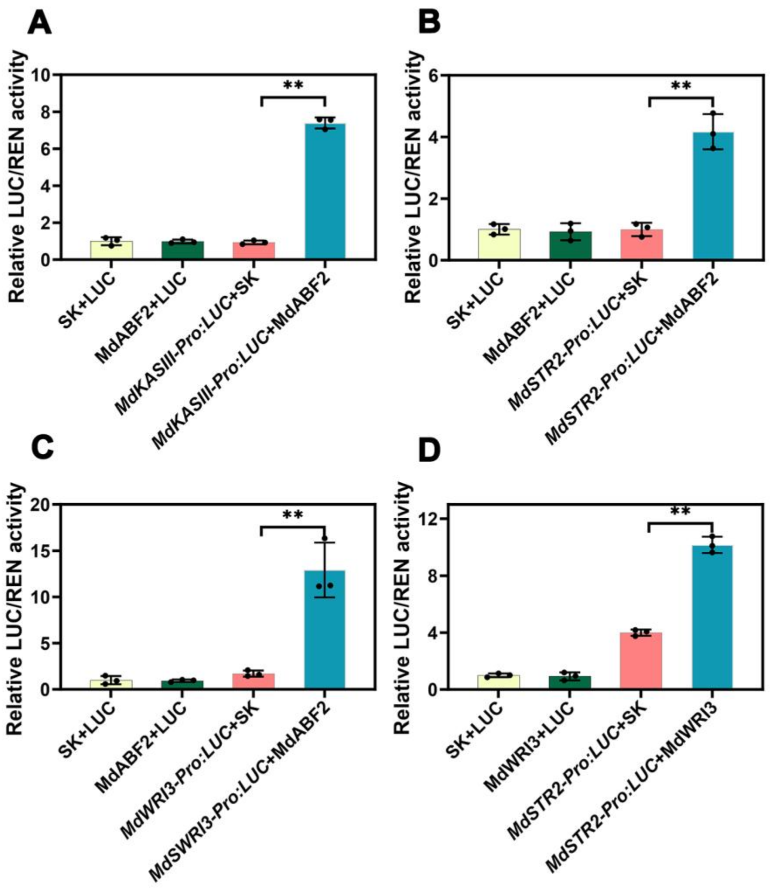

Supplemental Figure 18. Relative LUC/REN activity of reporter and effector vectors for the dual-luciferase assays via transient expression assays in tobacco. (A) Relative LUC/REN activity of co-expressing MdABF2 and *MdKASIII-Pro:LUC*. **(B)** Relative LUC/REN activity of co-expressing MdABF2 and *MdSTR2-Pro:LUC*. **(C)** Relative LUC/REN activity of co-expressing MdABF2 and *MdWRI3-Pro:LUC*. **(D)** Relative LUC/REN activity of co-expressing MdWRI3 and *MdSTR2-Pro:LUC*. Relative LUC/REN activity, with its activity in SK+LUC set as ‘1’. The bars represent the mean value ± SD (n = 3, representing independent biological replicates). Mixed samples from three transgenic tobacco lines were as one replicate. The asterisks indicate significant differences as assessed by one-way ANOVA. (two-sided Student's t-test; **P < 0.01). SK. 62-SK; LUC, pGreenII 0800-LUC.

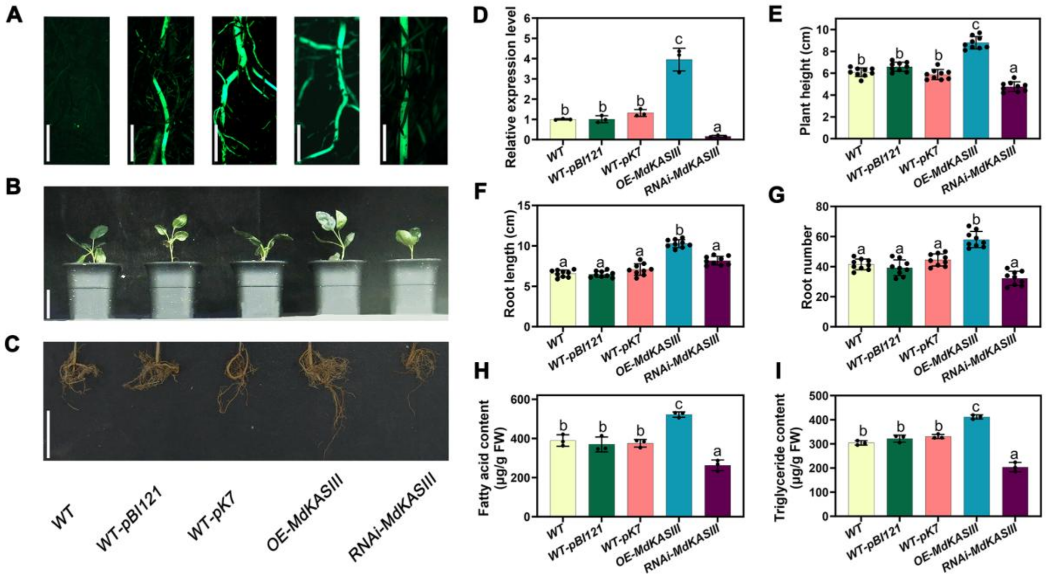

Supplemental Figure 19. The effects of changing the expression of *MdKASIII* in the roots of 60-day-old apple (*Malus hupehensis* Rhed) transgenic hairy roots not inoculated with AMF. Each construct contained a GFP cassette for screening the transformation. (A) Images of the transgenic root systems with green fluorescent protein (GFP). Scale bars, 1 mm. **(B)** Above-ground growth phenotypes of apple transgenic hairy roots. Scale bars, 5 cm. **(C)** Root growth phenotypes of apple transgenic hairy roots. Scale bars, 5 cm. **(D)** The mRNA relative expression levels of *MdKASIII* in the transgenic roots compared to the WT control (set as '1'). **(E)** The plant height of apple transgenic hairy roots. **(F)** The root length of apple transgenic hairy roots. **(G)** The root number of apple transgenic hairy roots. **(H)** The fatty acid content in the roots of apple transgenic hairy roots. **(I)** The triglyceride content in the roots of apple transgenic hairy roots. FW, fresh weight; WT, wild type; WT-pBI121, apple seedlings transformed with an empty overexpression vector containing the GFP tag (plasmid Binary Vector 121); WT-pK7, apple seedlings transformed with an empty RNA interference vector containing the GFP tag (pK7GWIWG2); OE-*MdKASIII*, *MdKASIII*-overexpressing root lines; RNAi-*MdKASIII*, *MdKASIII*-RNA interference root lines. (**D**, **H**, and **I)** The bars represent the mean value ± SD (n = 3, representing independent biological replicates). Mixed samples from three apple seedlings carrying transgenic hairy roots were as one replicate. (**E**, **F**, and **G)** The bars represent the mean value ± SD (n = 9, representing independent biological replicates). (**D**, **E**, **F**, **G**, **H**, and **I)** Different letters indicate significant difference (analysis of variance [ANOVA]), Duncan's multiple range test; $P < 0.05$).

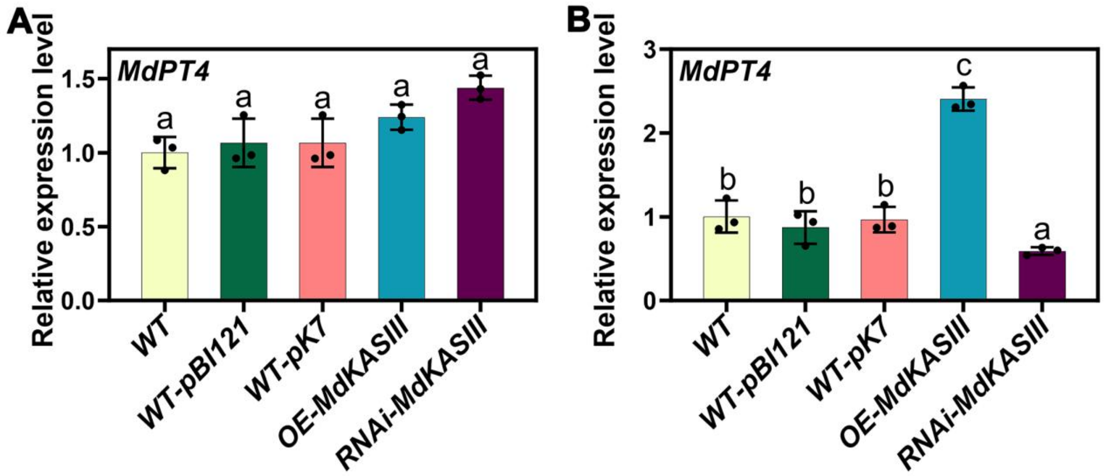

Supplemental Figure 20. Relative expression levels of *MdPT4* in the roots of 60-day-old apple (*Malus hupehensis* Rhed) transgenic seedlings. (A) The relative expression level of *MdPT4* based on qPCR in WT, WT-pBI121, WT-pK7, OE-*MdKASIII*, and *MdKASIII* apple transgenic hairy roots without *R*. *irregularis* infection of the transgenic roots. **(B)** The relative expression level of *MdPT4* based on qPCR in WT, WT-pBI121, WT-pK7, OE-*MdKASIII*, and RNAi-*MdKASIII* apple transgenic hairy roots with *R*. *irregularis* infection of the transgenic roots. Relative expression levels for each gene were obtained via the ddCT method, with its expression in WT set as '1'. The bars represent the mean value ± SD (n = 3, representing independent biological replicates). Mixed samples from three apple seedlings carrying transgenic hairy roots were as one replicate. Different letters indicate significant difference (analysis of variance [ANOVA]), Duncan's multiple range test; $P < 0.05$).

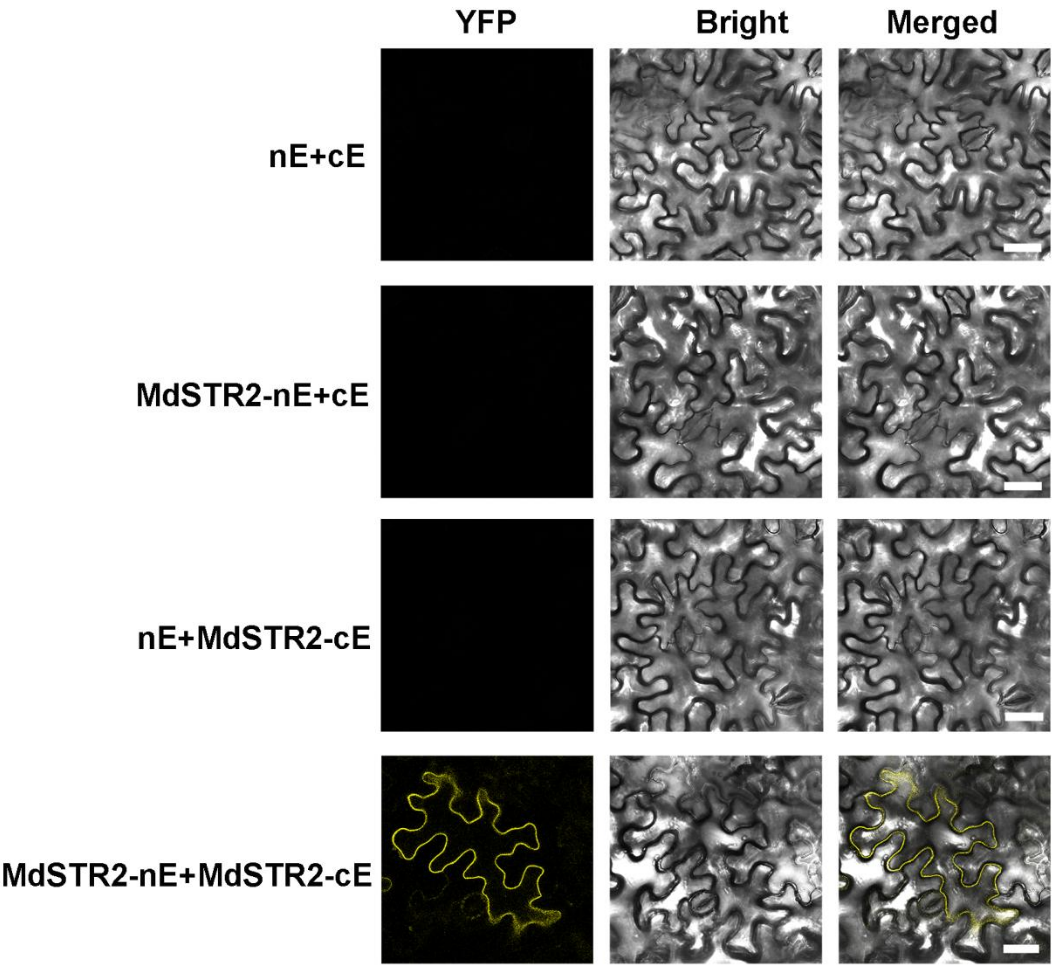

Supplemental Figure 21. Bimolecular fluorescence complementation (BiFC) assays indicated that MdSTR2 can form homodimer in apple. MdSTR2 was fused to the N-terminal fragment of YFP (nE). MdSTR2 was fused to the was fused to the C-terminal fragment of YFP (cE). Scale bars, 20 μm.

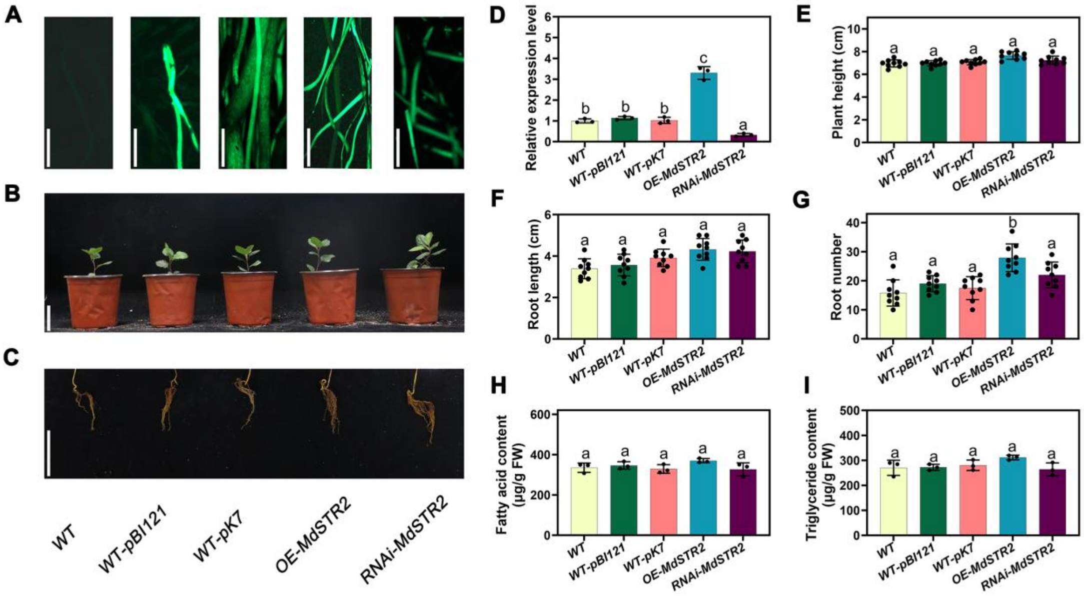

Supplemental Figure 22. The effects of changing the expression of *MdSTR2* in the roots of 60-day-old apple (*Malus hupehensis* Rhed) seedlings not inoculated with AMF. **Each construct contained a GFP cassette for screening the transformation.** **(A)** Images of the transgenic root systems of the apple seedlings with green fluorescent protein. Scale bars, 1 mm. **(B)** Above-ground growth phenotypes of apple transgenic hairy roots. Scale bars, 5 cm. **(C)** Root growth phenotypes of apple transgenic hairy roots. Scale bars, 5 cm. **(D)** The mRNA relative expression levels of *MdSTR2* in the transgenic roots of the apple seedlings compared to the WT control (set as '1'). **(E)** Plant height of apple transgenic hairy roots. **(F)** Root length of apple transgenic hairy roots. **(G)** Root number of apple transgenic hairy roots. **(H)** The fatty acid content of transgenic roots in the apple seedlings. **(I)** The triglyceride content of transgenic roots in the apple seedlings. FW, fresh weight; WT, wild type; WT-pBI121, apple seedlings transformed with an overexpressed empty vector containing the GFP tag (plasmid Binary Vector 121); WT-pK7, apple seedlings transformed with an empty RNA interference vector containing the GFP tag (pK7GWIWG2); OE-*MdSTR2*, *MdSTR2*-overexpressing root lines; RNAi-*MdSTR2*, *MdSTR2* -RNA interference root lines. (**D**, **H**, and **I)** The bars represent the mean value ± SD ($n = 3$, representing independent biological replicates). Mixed samples from three apple seedlings carrying transgenic hairy roots were as one replicate. (**E**, **F**, and **G)** The bars represent the mean value ± SD ($n = 9$, representing independent biological replicates). (**D**, **E**, **F**, **G**, **H**, and **I)** Different letters indicate significant difference (analysis of variance [ANOVA]), Duncan's multiple range test; $P < 0.05$).

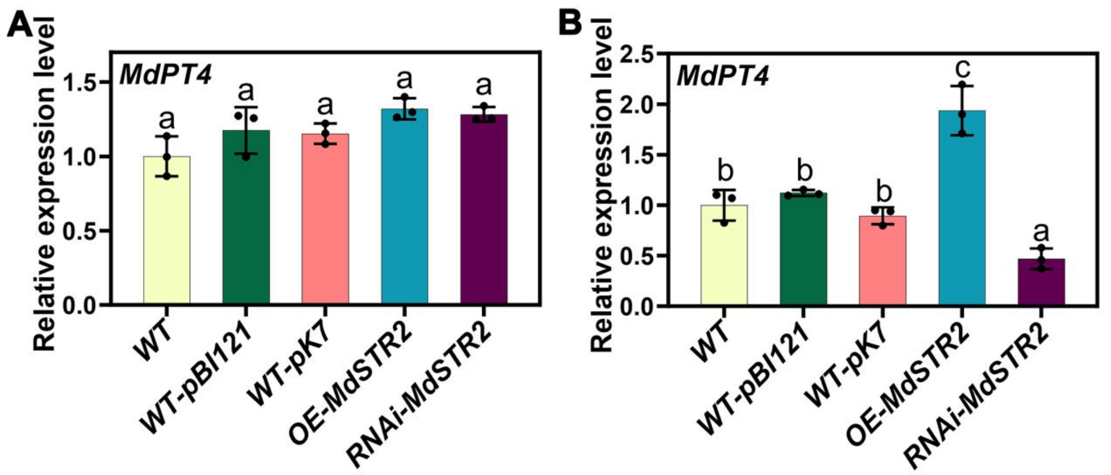

Supplemental Figure 23. Relative expression levels of *MdPT4* in the roots of 60-day-old apple (*Malus hupehensis* Rhed) transgenic seedlings. (A) The relative expression level of *MdPT4* based on qPCR in WT, WT-pBI121, WT-pK7, OE-*MdSTR2* and RNAi-*MdSTR2* apple transgenic hairy roots without *R*. *irregularis* infection of the transgenic roots. **(B)** The relative expression level of *MdPT4* based on qPCR in WT, WT-pBI121, WT-pK7, OE-*MdSTR2* and RNAi-*MdSTR2* apple transgenic hairy roots with *R*. *irregularis* infection of the transgenic roots. Relative expression levels for each gene were obtained via the ddCT method, with its expression in WT set as ‘1’. The bars represent the mean value ± SD (n = 3, representing independent biological replicates). Mixed samples from three apple seedlings carrying transgenic hairy roots were as one replicate. Different letters indicate significant difference (analysis of variance [ANOVA]), Duncan's multiple range test; $P < 0.05$).

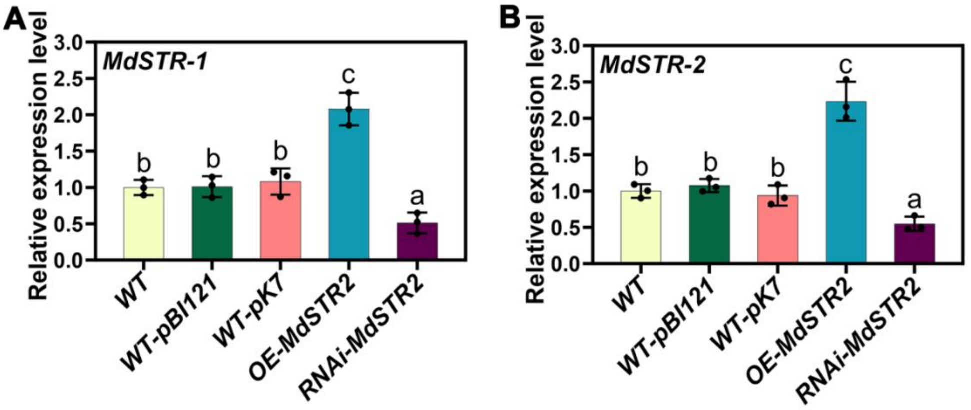

Supplemental Figure 24. Relative expression levels of *MdSTR* in the roots of 60-day-old apple (*Malus hupehensis* Rhed) transgenic seedlings with *R. irregularis* infection of the transgenic roots. (A) The relative expression level of *MdSTR-1* based on qPCR in WT, WT-pBI121, WT-pK7, OE-*MdSTR2* and RNAi-*MdSTR2* apple transgenic hairy roots. **(B)** The relative expression level of *MdSTR-2* based on qPCR in WT, WT-pBI121, WT-pK7, OE-*MdSTR2* and RNAi-*MdSTR2* apple transgenic hairy roots. Relative expression levels for each gene were obtained via the ddCT method, with its expression in WT set as '1'. The bars represent the mean value ± SD (n = 3, representing independent biological replicates). Mixed samples from three apple seedlings carrying transgenic hairy roots were as one replicate. Different letters indicate significant difference (analysis of variance [ANOVA]), Duncan's multiple range test; $P < 0.05$).
